# Biorefinery Approach for Aerogels

**DOI:** 10.3390/polym12122779

**Published:** 2020-11-24

**Authors:** Tatiana Budtova, Daniel Antonio Aguilera, Sergejs Beluns, Linn Berglund, Coraline Chartier, Eduardo Espinosa, Sergejs Gaidukovs, Agnieszka Klimek-Kopyra, Angelika Kmita, Dorota Lachowicz, Falk Liebner, Oskars Platnieks, Alejandro Rodríguez, Lizeth Katherine Tinoco Navarro, Fangxin Zou, Sytze J. Buwalda

**Affiliations:** 1 MINES ParisTech, Center for Materials Forming (CEMEF), PSL Research University, UMR CNRS 7635, CS 10207, 06904 Sophia Antipolis, France; daniel.aguilera-bulla@mines-paristech.fr (D.A.A.); coraline.chartier@mines-paristech.fr (C.C.); zoufangxin@iccas.ac.cn (F.Z.); 2 Faculty of Materials Science and Applied Chemistry, Institute of Polymer Materials, Riga Technical University, P.Valdena 3/7, LV, 1048 Riga, Latvia; sergejs.beluns@rtu.lv (S.B.); sergejs.gaidukovs@rtu.lv (S.G.); oplatnieks@gmail.com (O.P.); 3 Division of Materials Science, Department of Engineering Sciences and Mathematics, Luleå University of Technology, SE-971 87 Luleå, Sweden; linn.berglund@ltu.se; 4 Bioagres Group, Chemical Engineering Department, Faculty of Science, Universidad de Córdoba, Campus of Rabanales, 14014 Córdoba, Spain; eduardo.espinosa@uco.es (E.E.); a.rodriguez@uco.es (A.R.); 5 Department of Agroecology and Plant Production, Faculty of Agriculture and Economics, University of Agriculture, Aleja Mickieiwcza 21, 31-120 Kraków, Poland; agnieszka.klimek@urk.edu.pl; 6 Academic Centre for Materials and Nanotechnology, AGH University of Science and Technology, al. A. Mickiewicza 30, 30-059 Krakow, Poland; akmita@agh.edu.pl (A.K.); dorota.bielska@agh.edu.pl (D.L.); 7 Department of Chemistry, Institute for Chemistry of Renewable Resources, University of Natural Resources and Life Sciences, Vienna (BOKU), Konrad Lorenz Straße 24, A-3430 Tulln an der Donau, Austria; falk.liebner@boku.ac.at; 8 CEITEC-VUT Central European Institute of Technology—Brno university of Technology, Purkyňova 123, 612 00 Brno-Královo Pole, Czech Republic; Katherine.Tinoco@ceitec.vutbr.cz

**Keywords:** biomass, aerogel, lignocellulose, cellulose, nanocellulose, starch, chitosan, alginate, pectin, carrageenan

## Abstract

According to the International Energy Agency, biorefinery is “the sustainable processing of biomass into a spectrum of marketable bio-based products (chemicals, materials) and bioenergy (fuels, power, heat)”. In this review, we survey how the biorefinery approach can be applied to highly porous and nanostructured materials, namely aerogels. Historically, aerogels were first developed using inorganic matter. Subsequently, synthetic polymers were also employed. At the beginning of the 21st century, new aerogels were created based on biomass. Which sources of biomass can be used to make aerogels and how? This review answers these questions, paying special attention to bio-aerogels’ environmental and biomedical applications. The article is a result of fruitful exchanges in the frame of the European project COST Action “CA 18125 AERoGELS: Advanced Engineering and Research of aeroGels for Environment and Life Sciences”.

## 1. Introduction

Aerogels are dry, ultra-light, and highly porous polymer networks having a high internal pores’ surface area. They were first described in 1931 by S. Kistler who removed the liquid from a silica gel via supercritical CO_2_ drying to create a solid, porous network that kept its 3D structure [1]. He also described the preparation of aerogels from cellulose, gelatin, egg albumin, and agar, but their properties were not reported [2]. In the following decades, technological constraints severely limited aerogel research and development, and the field remained largely unnoticed. In 1970, S. Teichner revived aerogel research by describing a new preparation route for silica aerogels, which significantly reduced the time needed for aerogel production and improved aerogel properties [3]. The renewed interest of academic and industrial researchers led to several developments in aerogel science and technology in the 1980s and 1990s, including further improvement of silica aerogels’ properties and aerogel production at pre-industrial scale. Since aerogels may exhibit a thermal conductivity below that of air under ambient conditions, aerogels were mainly applied as thermal super-insulating materials in, e.g., the construction sector [4,5].

At the end of the 20th century and in the first two decades of the 21st century, aerogels based on synthetic polymers were developed, including polyamide [6], polyimide [7] and polyurethane [8]. At the same time, aerogels based on polysaccharides and proteins, so-called bio-aerogels, started to be investigated in a systematic manner. Today’s interest in bio-aerogels is in part due to the desire to use sustainable resources instead of fossil-based ones in order to reduce the environmental impact of the polymer industry and to realize a bio-based and sustainable society. In addition, bio-sourced polymers are often biocompatible as well as biodegradable and many exhibit anti-inflammatory and antibacterial effects [9,10]. As these properties are highly beneficial from a biomedical point of view, bio-aerogels are widely investigated for applications such as drug delivery [11], tissue engineering [12], wound dressings [13], and bio-sensing [14]. Thanks to their unique characteristics, including a high porosity, a low density and high specific surface area, bio-aerogels may also be used as food packaging [15], thermal insulation [16], catalysts and catalytic supports [17], as well as absorbents and adsorbents (e.g., for water purification) [18]. 

**Figure 1:**
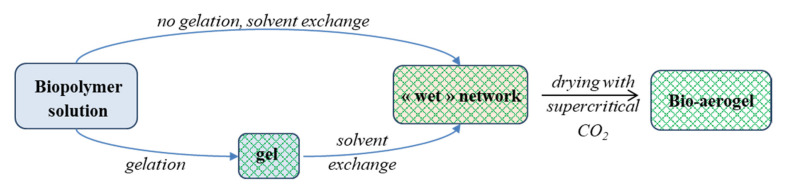
Schematic presentation of bio-aerogel preparation.

In general, bio-aerogels are prepared by polymer dissolution, gelation (in certain cases this step can be omitted), solvent exchange and drying with supercritical CO_2_ (Figure 1). This is fundamentally different from the preparation process of classical (e.g., silica) aerogels, which starts with the polymerisation of monomers. Whereas silica aerogels have been thoroughly investigated and correlations between preparation, structure and properties have been well established, bio-aerogels are recent materials, for which such correlations only begin to be deduced. Several excellent reviews have been published recently that underline the relevance of bio-aerogel research, including those focusing on biomedical applications [19], food applications [20], aerogel particles [21], water purification [22], and thermal insulation [23]. 

Even though the materials for bio-aerogels may be obtained from renewable sources, bio-aerogel preparation often consumes significant amounts of resources (e.g., solvents, non-solvents, CO_2_) and energy. Recently, researchers are increasingly focusing on the preparation of bio-aerogels via biorefinery approaches, which process biomass in a sustainable manner into a spectrum of marketable bio-based products [24]. An important motivation for the development and implementation of biorefinery approaches is the need for a secure and sustainable supply of feedstock that can address the growing demand for energy, fuels and chemicals [25]. Also, a reduction of fossil CO_2_ emissions and a revitalization of rural areas are the main drivers for the progressive replacement of oil refinery by biorefinery approaches. To realize this transformation, it is necessary to integrate different and complex processes in the same facility, achieving an efficient use of resources and ensuring sustainability of the overall process.

All biorefineries have the same generic scheme of operation, such as the fractionation of components and their use or transformation separately. However, the high variety of different biomass included in the biorefinery concept, makes it necessary to have specific technology and processes depending on the raw material used and the desired product. The IEA Bioenergy developed a biorefinery classification system according to biomass feedstock, the platform, the products, and the processes used [26]. These biomass feedstocks feature grasses, starch (wheat and corn) and sugar (beet and cane) crops, lignocellulosic crops and residues, oil crops, aquatic biomass (algae and seaweeds) and organic residues [27]. Biorefinery platforms are the intermediate products derived from biomass that will be used for conversion into final products. Several types of platforms can be defined, such as syngas, C5 and C6 sugars (from lignocellulosic biomass), lignin, oil (from oil crops and algae), and biogas. The processes used for the biomass fractionation and platform products conversion are divided into four groups: (i) mechanical/physical, (ii) biochemical, (iii) chemical, and (iv) thermochemical. In addition, depending on the type of outputs produced, biorefineries are classified into systems focused on the production of energy or of material [27].

This review highlights, for the first time, biorefinery approaches that have been used, or attempted to use, for the preparation of bio-aerogels, also focusing on processing-structure-properties relationships. This manuscript starts with a general description of sustainable processing options for the preparation of bio-aerogels. Subsequently, several case studies are presented including examples of bio-aerogel properties and a brief overview of applications. Specifically, aerogels prepared from lignocellulose, marine polysaccharides, chitosan, pectin, starch, proteins, and organic acids are discussed. Lastly, the authors’ vision on the current challenges and future prospects in this rapidly developing area of research is presented. The goal of this review is not to provide a detailed description of aerogel structure and properties from each type of biomaterial. Indeed, each deserves a separate review article. Our objective is to present the reader an overview on how renewable resources can be used for making aerogels, and what the prospects are in terms of using the biorefinery approach. When possible, we will focus on biomedical and environmental applications of bio-aerogels.

To conclude this introduction, it is important to present our definition of aerogel, which will be used throughout this manuscript, since the term ‘aerogel’ is not standardized. In the IUPAC Gold Book, an aerogel is described as ‘a gel comprised of a microporous solid in which the dispersed phase is a gas’ [28]. This description is rather restrictive as it focuses on materials with pore size below 2 nm, thus excluding even classical silica aerogels which are mesoporous. Based on previous literature, the following definition for aerogels is used in the present review: an aerogel is an open pores, solid network, exhibiting a high porosity (at least 90%), a high specific surface area (for this criterium no official convention exists [29] but we consider “high” to be at least 100 m^2^/g) and a nanostructured morphology (mainly mesoporous with small macropores). This definition excludes other porous materials such as foams/sponges/cryogels, gels/hydrogels and membranes. Sponges, foams, and cryogels have very large pores, thereby displaying a low specific surface area, gels/hydrogels contain a solvent in the pores and membranes may not be of very low density. Freeze-dried biobased materials (often called “cryogels”) will be considered if they present a high specific surface area or provide important results helping the interpretation of the properties of aerogels.

## 2. Green Chemistry and Sustainability in the Manufacture of Bio-Based Aerogels

Never before in human history have so many people populated our planet. Never before has the pace at which global fossil and mineral resources, formed over millions of years far before human beings populated our earth, been depleted so fast as it is today. Never before have intelligent life forms succeeded to create an ecological scenario that bears high risk to irreversibly unhinge global equilibria which are supposed to be their natural foundation. It is therefore high time for serious changes targeting a more responsible sharing and exploitation of natural resources, the implementation of smart processing technologies avoiding hazardous chemicals and environmental pollution, and the development of tailor-made functional materials with better end-of-life control.

Based on an increasing awareness of the fact that our fossil resources (oil, gas, and coal) will be depleted within a time span that will be probably not much longer than that elapsed since the industrial revolution, consent seems to exist in broad groups of our society that renewable resources inevitably need to regain an important role in the near future. This has led to numerous projects and activities launched in the last decade which—at least some of them—paved the way towards a more bio-based economy. Even though renewables are still being used mainly for energy production (e.g., wood pellets, biogas, bioethanol, biodiesel), it is to assume that in the near future renewables will primarily serve as feedstock for chemicals and materials. While more efficient energy providing technologies based on wind, water, solar, or nuclear energy exist, or will become available soon, no alternatives are in sight for chemical/material utilization of renewable sources. 

Besides the unavoidable necessity for rethinking the predominantly wasteful and careless way we have been utilizing our natural resources in the last centuries, changes in current technologies are urgently required too, if we should be aiming to hand over a largely healthy ecosphere to future generations. This includes not only a more efficient use of energy (which is mostly given in the private production sector), but in particular, improved product life cycle efficiency, reduction of process- and/or usage-related environmental pollution, and end-of-use recyclability or biodegradability. 

The increasing implementation of the principles of green chemistry in the design of new products and product technologies can be regarded an important countermeasure against the current complex of environmental problems and a significant turn towards a more ecofriendly use and processing of natural resources. Having its roots in the US 1990 Pollution Prevention Act and the subsequently initiated source reduction program of the Environmental Protection Agency (EPA), Green Chemistry has developed into a philosophy that encompasses all areas of chemistry and is aiming to develop solutions for real-world environmental problems [30]. Since chemistry, from a simplified point of view, can be seen as a sequence of educts, process conditions and products, the efforts being made under the umbrella of green chemistry target the entire chain from responsive selection and consumption of the source material, implementation of environmentally friendly and energy-efficient processes till the creation of products with tailor-made properties and low intrinsic hazard for humans and our ecosphere. This includes the call for an increasing use of renewable resources, the avoidance of chemical derivatives, safer solvents, optimization of reaction conditions and yield using catalysts, and prevention of waste (details of green chemistry’s 12 principles can be found elsewhere [30]).

The intriguing properties of aerogels, well known as silica-based pioneer materials in this field, along with the creativity of material scientists in exploring new sources for more and more new applications has literally triggered a boom in aerogel research [31]. This applies also for the still comparably small fraction of bio-based aerogels [31,32]. There is a cautious but justified optimism that some of the recently developed bio-aerogels will step out of lab- or pilot scale development to join the market in the near future. 

Many papers claim in their headings, abstracts or conclusions successful implementation of green approaches or development of respective sustainable materials. However, a closer look sometimes suggests that terms like “green”, “sustainable”, “environmental-friendly”, or “bio-based” are used in a somewhat too superficial, inflationary, or attention-attracting way. A detailed analysis of the major stages of aerogel manufacture might help to initiate a somewhat more sensible discussion of this very complex issue.

With regard to sustainability and alignment with the principles of green chemistry it should be noted that bio-aerogels meet at least two criteria: bio-aerogels are intrinsically ultra-lightweight, optimized with regard to weight-to-stiffness ratio which can greatly reduce source consumption, and they are derived from renewables. Their low bulk densities and attainable low thermal heat conduction, as imparted by sophisticated open-pores network architectures, can directly translate into significant energy savings, if they are used in the transportation or building sectors. Respective promising materials from pectin [33], cellulose [34,35], starch [36], or alginate [37], partially in combination with inorganic networks composed of silica [38] or zeolites [39], have been presented in the last years. However, remaining issues including reduction and recycling of solvents, optimization of solvent extraction by scCO_2_, and development of measures efficiently suppressing water vapor sorption, microbial degradation or ignition of the bio-based aerogels still need to be solved prior to commercialization. 

At a first glance, bio-based aerogels seem to meet a series of even more criteria of green chemistry. These include: (i) their biodegradation to innocuous compounds, hence preventing waste, (ii) design of safer products posing no hazard or health risk to both producers and consumers, and (iii) maximizing of atom economy ensuring that the final products contain the highest possible proportion of the respective starting material. However, these aspects require a more in-depth discussion. 

It is true that the aforementioned criteria are commonly met for the manufacture of aerogels from pristine purified biopolymers. It is important, however, not to forget that a series of pretreatments is commonly required to separate and purify the respective biopolymers from real-world renewable resources. These processes require energy, chemicals, equipment, create byproducts and comply, hence, not necessarily with all goals of the green chemistry philosophy. Cellulose aerogels, for example, are almost exclusively made from highly purified dissolving cellulose which, in turn, is the product of a rather energy and chemical intensive wood pulping and bleaching process. Other biopolymers require similar pretreatments. For example, a vast majority of lignin produced worldwide is a byproduct of kraft-type wood pulping and is isolated from the hot pulping “black” liquor by two-step precipitation using, for example, carbon dioxide and mineral acids. Further steps include filtration and, depending on the applied technology, repeated purification measures aiming to reduce the content of inorganics. Similarly, chitosan and alginates require pretreatments at an expense that should not be underestimated. The production of alginates starts with ship-based harvesting of brown algae from the sea bottom, followed by washing, drying, milling, sodium hydroxide extraction, repeated filtration, and precipitation. Chitosan stands at the end of a similarly lengthy process starting with washing and decalcification of crab shells after food service, proceeds via deproteinization and discoloration, and ends with partial deacetylation by either enzymatic (chitin deacetylase) or chemical treatment (hot HCl). 

It was stated above that processing of pristine biopolymers into aerogels largely preserves the biodegradation capabilities of the source materials to innocuous compounds and affords products at high atom economy since losses are negligibly small. This applies for processes such as molecular dispersing dissolution/coagulation (e.g., cellulose [40,41,42]), heat-induced gelatinization/retrogradation (e.g., starch [43,44]), or osmotic concentration of aqueous dispersions of cellulose nanofibrils [34] which either employ direct freeze-drying or scCO_2_ drying after preceding solvent exchange (typically from water to ethanol). However, it does not necessarily apply for other types of bio-based aerogels, such as (i) those made from biopolymer derivatives, (ii) aerogels formed by covalent or ionic crosslinking using bi- and multifunctional organic or inorganic reagents, (iii) those containing interpenetrating networks of secondary inorganic or organic constituents and (iv) hybrid materials carrying covalently or physically bonded bioactive, photoluminescent, catalytically active inorganic compounds or (nano)particles on their large internal surfaces. For those above-mentioned materials which nowadays represent a high percentage of bio-based aerogels, the efficiency of derivatization (atom economy), toxicity of used chemicals, whereabouts of byproducts, consumption and recycling of solvents, biodegradability, and health aspects of metabolites need to be included in respective life cycle and sustainability assessments. 

Cellulose mass products like microcrystalline cellulose (E 460), methyl cellulose (E 461), hydroxypropyl cellulose (E 463), hydroxypropyl methyl cellulose (E 464) or sodium carboxy methylcellulose (E 466) are of low toxicological concern (showing no genotoxicity, carcinogenic effects, short-term subchronic or chronic toxicity at relevant dosage [45]) and have been therefore approved as food additives. However, there is currently a strongly increasing range of biopolymer derivatives that are researched for non-food aerogel applications but of largely unknown health risk and biodegradation characteristics. This includes those materials that are subjected to oxidation [35,46], acetylation [47], carbamylation [48], hydroxypropylation [49] or polyethyleneimine grafting of cellulose [50], oxidation of hemicellulose [51], carboxymethylation of chitosan [52], methylolation [53] and amination of lignin [54], or the amidation of pectin [37]. Even for cellulose nanoparticles safety aspects have not always been conclusively investigated. Recently it has been shown that the latter can be taken up by embryonic zebrafish (*Danio rerio*) during development, a frequently used vertebrate model of toxicity. While surface chemistry had a minimal influence on the overall toxicity of nanocellulose materials, aspect ratio and type of defibrillation were of greater importance [55]. Cellulose esters are also not entirely innocuous as evident from the example of sebacic acid modified cellulose which has been recently shown to have renotoxic effects and increase the susceptibility of kidney toxicity after subacute exposure [56].

Crosslinking of biopolymers, insertion of percolating networks from secondary constituents or incorporation of nanoparticles, photosensitizers or other functional groups render life cycle, risk, and sustainability assessments much more complex. Two examples might illustrate this. The first is temperature-sensitive cellulose nanofibril (CNF) microspheres for controlled drug release: CNF beads crosslinked with polyamide-epichlorohydrine were prepared using a spray freeze-drying method [57]. In this case, a solution of *N*-isopropyl acrylamide, *N,N*-methylenebisacrylamide and initiator potassium persulfate were loaded into CNF spheres to accomplish concurrent polymerization and crosslinking which was performed in a mixture of kerosene and the emulsifier Span-85. While for the first part of preparation high-pressure equipment and liquid nitrogen is required, polymerization needs inert atmosphere, vigorous stirring, heating, multiple water/acetone washing steps and final freeze-drying. The second example concerns featherlight, mechanically robust cellulose ester aerogels for environmental remediation [58]: here, cellulose diacetate is first synthesized and afterwards subjected to crosslinking with pyromellithic anhydride. Aiming at high oil sorption capacities (achieved up to 112 g/g), the materials were subjected to chemical vapor deposition of trichlorooctylsilane. The ideas behind the described works are undoubtedly great, the targeted applications require adaption and modification of the source material. What remains is to critically assess “profitability”, sustainability, and biodegradability of the prepared materials. 

The conversion of gels into “cryogels” (via freeze-drying) or aerogels (via drying with supercritical CO_2_) is another issue that may negatively impact profitability and sustainability. Drying in ambient conditions usually leads to the formation of strong capillary forces resulting in the collapse of the gel network. Freeze-drying is regarded as a desirable approach, however dry network properties strongly depend on the freezing protocol, used pore liquids, addition of cryoprotectants and cooling media. Supercritical drying, in particular with carbon dioxide (scCO_2_), is regarded as the gentlest drying approach since the morphology of the gel network can be more or less preserved. However, owing to their hydrophilicity, bio-aerogels are frequently prepared via the respective hydrogel state. Since water is not miscible with scCO_2_, gradual solvent exchange steps from water to an organic solvent (ethanol, acetone, DMSO) miscible with scCO_2_ are required prior to drying. It should also be noted that scCO_2_ drying requires a certain high pressure and is a batch process; the development of continuous scCO_2_ drying approaches are highly requested. The first promising results for countercurrent scCO_2_ drying of alginate aerogel particles have been recently presented [59]. The next step is to try to apply this approach for the manufacture of aerogel monoliths.

The research on aerogels, and in particular on bio-aerogels, is continuously growing. Despite its attractiveness, the used bio-based matter does not automatically imply economic attractivity, sustainability, good biodegradability, no waste, or pollution or zero health risk. Consideration of all points together and applying a life cycle analysis are a “must” for the future development of bio-aerogels.

## 3. Case Studies

This part presents an overview of the most important natural feedstocks that can be used for making bio-aerogels. Each section starts with a brief presentation of the natural polymer followed by examples of aerogels made from it and of their potential applications.

### 3.1. Aerogels from Lignocellulose

Lignocellulosic biomass is the most abundant renewable resource on the earth. Lignocellulose is produced by nature via photosynthesis that uses solar radiation and CO_2_. Till now, materials based on lignocellulose, and in particular, on cellulose, were not considered for high-tech applications or cutting-edge research. The reason is that for thousands of years lignocellulose was used for rather low-cost bulk products such as, for example, textile and paper. The situation changed in the 20th century with the development of cellulose chemical modifications and use of cellulose derivatives as additives and viscosity modifiers in food, cosmetics, pharmaceutics, paints, and also as thermoplastic polymers. A surge of interest to cellulose occurred at the beginning of the 21st century with the advancements in nanocellulose, opening new and unexpected applications. One example is cellulose-based aerogels [60,61,62]. In parallel, the research on lignin and hemicellulose also suggested new uses of these components mainly as low molecular weight additives. 

**Figure 2:**
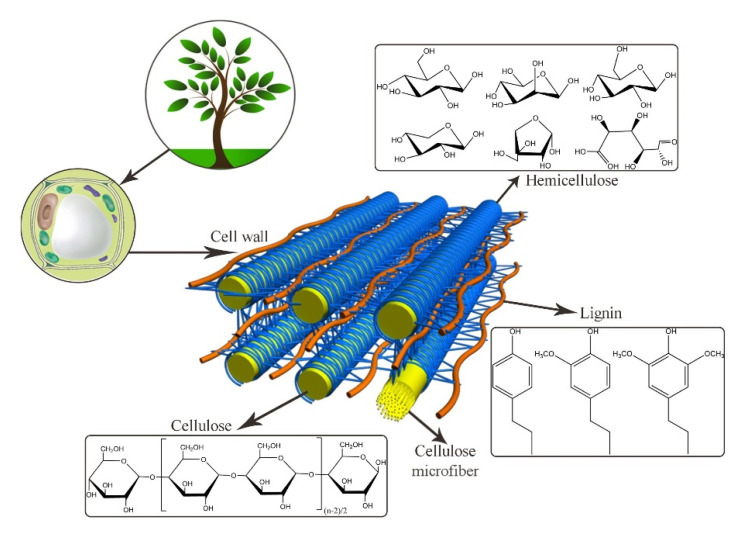
Chemical composition of the lignocellulosic matrix, adapted from [63]. Reprinted from Electrochimica Acta, 247, Song, A.; Huang, Y.; Liu, B.; Cao, H.; Zhong, X.; Lin, Y.; Wang, M.; Li, X.; Zhong, W., Gel Polymer Electrolyte Based on Polyethylene Glycol Composite Lignocellulose Matrix with Higher Comprehensive Performances, 505-515, Copyright 2017, with permission from Elsevier.

This section is devoted to aerogels based on lignocellulose, focusing on using all main components of this biomass: cellulose, lignin, and hemicellulose (Figure 2). First, two types of cellulose aerogels will be considered: cellulose I aerogels based on nanocellulose and cellulose II aerogels made via dissolution-coagulation route. The main difference between these two types of cellulose-based aerogels is that for the former the starting matter is a suspension of nanofibers (cellulose is not dissolved), while for the latter cellulose undergoes a dissolution step. Next, aerogels based on lignin, on hemicellulose and more or less entire lignocellulose will be presented. Finally, functionalization as a tool to enhance lignocellulose aerogel properties for environmental and life science applications will be discussed.

#### 3.1.1. Aerogels from Cellulose

**Figure 3:**
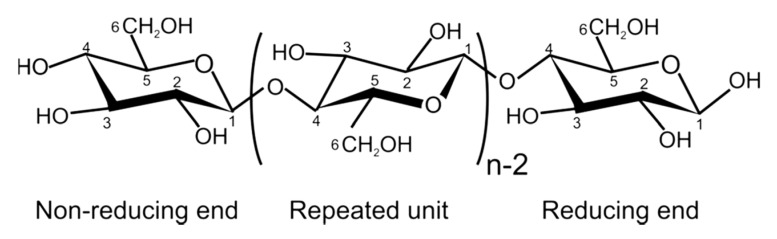
Structural formula of cellulose [64]. Reprinted by permission from [Springer] [Cellulose] [Glucose, not cellobiose, is the repeating unit of cellulose and why that is important; A. D. French, 24, 4605-4609] COPYRIGHT 2017.

Cellulose is a linear homopolymer based on D-glucopyranose connected by (1→4)-β linkages (Figure 3). It is the most abundant natural polymer and is thus an important source of sustainable materials. Cellulose is a polymer with structural functions; it can be found in wood and numerous plant sources as well as in fungi and algae. Wood contains about 40–50% of cellulose, higher amounts can be found in plants such as cotton (95%), hemp (75–80%), flax (70–80%), and ramie (70–75%). Certain bacteria produce ultra-pure high molecular weight and highly crystalline bacterial cellulose.

“Cellulose” exists in different forms and terminology is a bit hectic because of the long history of using cellulose-based materials in various applications:

- It is a linear polymer as shown in Figure 3.
- Due to the numerous intra- and intermolecular hydrogen bonds, cellulose can be organised in crystals or be less ordered (amorphous). Crystal forms, allomorphs, also can be different. The majority of cellulose is organised in allomorph called “cellulose I” existing in native celluloses (plants, wood, bacteria). Next is “cellulose II” which is cellulose precipitated (or coagulated, or regenerated) from a solution or obtained by a treatment (swelling) in strong alkali (mercerisation). A detailed review on cellulose solvents can be found in [65]. Other cellulose allomorphs, cellulose III and IV, are obtained under special treatments.
- Cellulose macromolecules can be organized in “nanocellulose” which can be in the form of: (a) flexible nanofibers (cellulose nanofibers, CNF) and (b) crystals or whiskers (cellulose nanocrystals, CNC). Bacterial cellulose is also one of the types of nanocellulose.
- Microcrystalline cellulose (MCC) consists of highly crystalline cellulose I particles of few tens of microns in length and low aspect ratio. MCC often serves as a starting matter of cellulose II based aerogels as it is high purity low molecular weight cellulose which is rather easy to dissolve.
- Natural fibers extracted from wood or plants are often called “cellulose fibers” despite that they contain hemicelluloses, lignin and other natural components (waxes, pectin and inorganic molecules). The composition of natural fibers strongly depends on the type of plant or wood from which they are extracted and on the extraction steps (for example, delignification). “Cellulose fibers” can also be called “pulp” or “pulp fibers”; in the latter cases this concerns fibers extracted from wood.
- Finally, “cellulosic polymers” may be used to name cellulose ethers and esters. Chemical modification of cellulose leads to completely different polymer properties. For example, contrary to cellulose polymer, cellulose ethers can be water-soluble (for example, carboxymethyl cellulose) and cellulose esters can be thermoplastic (for example, cellulose acetate).

Various raw materials can be the source of fibers containing cellulose: wood, non-wood (plants, bacteria) and recycled paper. First, fibers should be extracted from the bulk (for example, from wood) and then treated and/or purified to different extent. The extraction route depends on the type of source and fiber application: for example, plant fibers such as flax and hemp can be used to reinforce plastics, and fibers from wood for making paper and “dissolving pulp”, the latter to spin fibers via dissolution and make textile. Fibers from recycled paper are used to make tissue paper, newspaper and cardboard. 

Wood is one of the main sources of natural fibers. The latter are extracted using various methods (chemical, mechanical, chemomechanical) combined with various treatments (bleaching, refining) to remove lignin, hemicelluloses, target certain cellulose molecular weight and/or perform functionalisation. The main goal is to remove lignin, which in turn can be the source of energy, if burned, or of aromatic molecules. 

In the view of making cellulose-based aerogels, two options will be considered: from nanocellulose and via dissolution-coagulation route. For the case of nanocellulose, not only the topology of the nanofibers is of primary importance but also their composition, as nanocellulose may contain lignin and hemicellulose (case of CNF), and also have various charges on their surface depending on the treatment. When aerogels are made from dissolved cellulose, usually either microcrystalline cellulose or dissolving pulps are used. The presence of other components is negligible, and it is cellulose molecular weight that governs the properties of the solution. 

##### Cellulose I Based Aerogels

**Figure 4:**
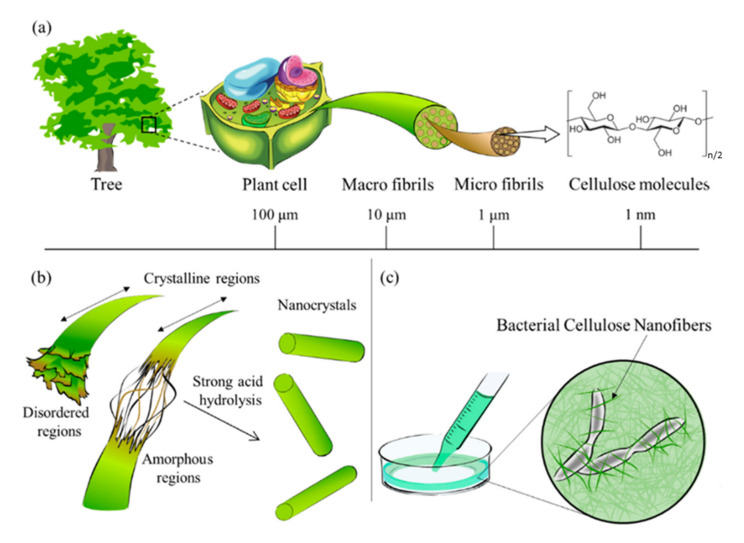
(**a**) Hierarchical structure from the meter to the nanometer scale (cellulose nanofibers) of a natural fiber contained in the plant cell wall; (**b**) schematic diagram of the isolation of cellulose nanocrystals by strong acid hydrolysis and (**c**) bottom-up production of bacterial cellulose [68].

Figure 4 shows the hierarchical organisation of natural (here, wood) fibers, from macro- to nano-scale. The nanometric size of nanocellulose confers additional characteristics such as a large specific and reactive surface [66]. Individual CNF are very strong, with Young’s modulus being 138 GPa and tensile strength 2–3 GPa [67]. 

Cellulose nanofibers are isolated from natural fibers via delamination, i.e., strong mechanical treatment [69]. To facilitate its effectiveness, various pre-treatments are used; the most common are enzymatic hydrolysis, 2,2,6,6-tetramethylpiperidine-1-oxyl (TEMPO) mediated oxidation, cationization, and mechanical refining [70,71,72,73]. Once pre-treated, the fibers are subjected to mechanical treatment for nanofibrillation which may consist of a high-pressure homogenization, microfluidization or ultrafine grinder process [74]. This type of nanocellulose contains crystalline and amorphous cellulose regions and has a diameter of 10–20 nm and several microns in length. Depending on the preparation route, CNF may contain different amounts of hemicellulose and may contain some lignin.

Cellulose nanocrystals, also known as cellulose whiskers, are rod-like entities, they are obtained from the acid hydrolysis of natural fibers. Acid hydrolysis (concentrated acids (60–70%) at temperatures around 45–70 °C during 15–45 min) degrades the amorphous regions of the fibers leading to the isolation of the crystalline regions [75,76]. These nanocrystals are composed of pure cellulose with a high crystallinity (54–88%) and present diameters between 2 and 20 nm and lengths of 100–500 nm [77,78].

Bacterial cellulose (BC) is a type of nanocellulose obtained from the “assembly” by bacteria of low molecular weight sugars into high molecular weight cellulose [79]. The main microorganism used to obtain BC is *Gluconacetobacter xylinus*. Other species are genus Agrobacterium and Rhizobium [80,81,82]. Unlike the other types of nanocellulose, BC is produced in pure form, it does not contain lignin, hemicellulose, pectin, or any other component of the lignocellulosic matrix. It is presented in the form of nanofibers with an average diameter of 3–10 nm and lengths of few micrometers. 

Due to a very high aspect ratio of nanocelluloses, especially of CNF and BC, they form hydrogels at low polymer concentrations, around few wt% and below [83]. Surface charge also plays an important role in gel formation and structure. For example, some CNC and CNF suspensions may contain anisotropic zones because of self-assembly. Due to the differences in the chemical composition and morphology of nanocelluloses, their aerogels will be separated into CNF-aerogels, CNC-aerogels and BC-aerogels.

##### CNF-Aerogels

Because of the very low concentration of solid matter in CNF hydrogels, the corresponding aerogels are of very high porosity, often above 99%. Specific surface area depends on the drying method. If using drying with supercritical CO_2_ the surface area can be up to 600 m^2^/g (see, for example, [35,46]). 

CNF concentration and surface charge play an important role in aerogel properties. Martoïa et al. studied the properties of freeze-dried CNF based on fibrils that underwent TEMPO-oxidation or enzymatic hydrolysis resulting in different surface charge [84]. Self-standing foams with low shrinkage were obtained when cellulose concentration was above the percolation concentration. The latter was around 0.2 wt% for TEMPO-oxidised CNF vs around 1 wt% for an enzymatically pre-treated one [84]. In general, the shrinkage of CNF-based gels during the preparation of aerogels is within 20% which is lower than that of other bio-aerogels (see the next sections).

**Figure 5:**
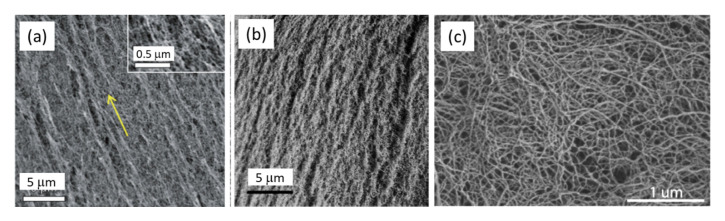
SEM images of the morphology of (**a**) TEMPO oxidized CNF (adapted with permission from Kobayashi, Y.; Saito, T.; Isogai, A. Aerogels with 3D Ordered Nanofiber Skeletons of Liquid-crystalline Nanocellulose Derivatives as Tough and Transparent Insulators. Angewandte Chemie International Edition 2014, 53, 10394–10397 [46], (**b**) 2,3-dicarboxyl CNF aerogel (adapted with permission from Plappert, S.F.; Nedelec, J.; Rennhofer, H.; Lichtenegger, H.C.; Liebner, F.W. Strain Hardening and Pore Size Harmonization by Uniaxial Densification: A Facile Approach Toward Superinsulating Aerogels from Nematic Nanofibrillated 2, 3-Dicarboxyl Cellulose. Chemistry of Materials 2017, 29, 6630–6641 [35]. Copyright (2017) American Chemical Society) and (**c**) enzymatically pre-treated TEMPO oxidized freeze-dried from tert-butanol CNF [85] (Reprinted from Composites Science and Technology, 71, Sehaqui, H.; Zhou, Q.; Berglund, L. A., High-porosity aerogels of high specific surface area prepared from nanofibrillated cellulose (NFC), 1593–1599, Copyright 2011, with permission from Elsevier).

To obtain high specific surface area (above 100 m^2^/g) of CNF-based porous materials, supercritical drying can be replaced by freeze-drying from tert-butanol or its mixtures with water. For example, surface area around 280 m^2^/g was obtained for enzymatically and TEMPO-oxidised CNF in which water in hydrogels was replaced by tert-butanol [85]. By optimising the composition of water/tert-butanol and thus improving the dispersion of CNF, the surface area was 320 m^2^/g [86]. However, the highest specific surface area of CNF-based aerogels, 400–600 m^2^/g, was obtained for CNF with charged surfaces and dried with sc CO_2_, either from TEMPO-oxidised CNF or after periodate/chlorite treatments resulting in 2,3-dicarboxyl CNF [35,46]. The comparison of the morphologies of supercritically and freeze-dried for tert-butanol CNF aerogels is shown in Figure 5.

No clear trend of specific surface area of CNF-based aerogels vs density has been reported till now: Kobayashi et al. showed no influence of density (and of CNF concentration in the initial gel) on aerogel surface area, it varied within 500–600 m^2^/g for densities from around 8 to 30 mg/mL [46]. No trend was observed for CNF aerogels based on 2,3-dicarboxyl CNF: density was varied by compressing aerogels and surface area varied from 400 to 590 m^2^/g [35]. For CNF foams obtained from TEMPO-oxidised freeze-dried Pickering emulsions, the surface area decreased from 65 to 15 m^2^/g with density increase from 0.012 to 0.03 g/cm^3^, respectively [87]. 

**Figure 6:**
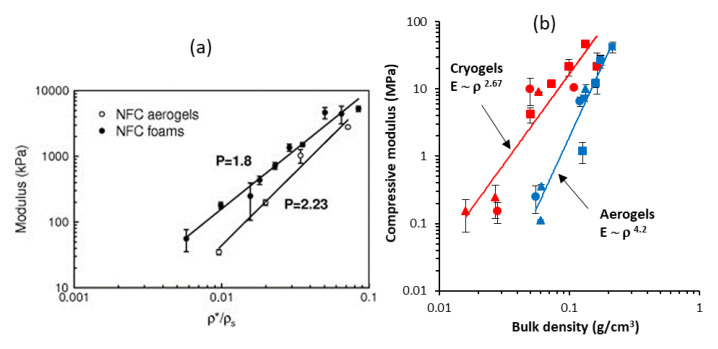
Compressive modulus as a function of (**a**) relative density for aerogels and foams based on CNF [85] (Reprinted from Composites Science and Technology, 71, Sehaqui, H.; Zhou, Q.; Berglund, L.A. High-porosity aerogels of high specific surface area prepared from nanofibrillated cellulose (NFC), 1593–1599, Copyright 2011, with permission from Elsevier) and (**b**) as a function of bulk density for aerogels and cryogels (or foams) based on cellulose II [89].

The influence of density on the compression modulus of CNF-based aerogels is not very clear either. Few works demonstrated a linear dependence of compressive modulus on material density [35,46] contrary to the power law known for the majority of aerogels and foams [88]. An interesting comparison of the mechanical properties under the uniaxial compression was made by Sehaqui et al. [85] (Figure 6): they showed that for freeze dried CNF foams the modulus is higher and the exponent P in the power law (P = 1.8) is lower as compared to aerogels obtained via supercritical drying (P = 2.23); the starting gel was enzymatically pre-treated CNF. An increase in aerogel density from 15 to 105 kg/m^3^ resulted in the increase of Young’s modulus from 34.9 to 2800 kPa, strength from 3.20 to 238 kPa and energy absorption from 10.8 to 720 kJ·m^3^, respectively [85]. Higher modulus and lower exponent were also reported for cellulose II cryogels as compared to aerogels from the same formulation [89]. The reason could be thicker pore walls in freeze-dried materials. Power law of the modulus vs density was also reported for freeze-dried CNF: the exponent was 2.29 for TEMPO-oxidised CNF and 3.11 for enzymatically pre-treated ones. The difference was explained by higher aspect ratio and stronger colloidal stability of TEMPO-oxidised CNF [84].

CNF-based aerogels also exhibit low thermal conductivity and acoustic absorption ability which allow using them as insulation materials. Low thermal conductivity is due to low density and small pores sizes, the latter providing the Knudsen effect to work. Depending on the density, aerogels thermal conductivity varied between 0.04 and 0.018 W·m^−1^·K^−1^ [46]. Low thermal conductivity was also reported by Chen et al.: the lowest values, around 0.014 W/m·K were obtained for CNF pre-treated either via TEMPO oxidation or via strong sulfuric acid [90]. While Plappert et al. et reported that compression decreased pore dimensions and thus decreased thermal conductivity due to the Knudsen effect [35], Chen et al. observed the opposite phenomenon, probably, because of a stronger input of the skeletal density [90]. For acoustic absorption properties, the CNF-based aerogels present a higher absorption ratio compared to particleboard and plywood. The sound absorption was poor at low frequencies (<1000 Hz) but increased up to 55–57% at higher frequencies (>4000 Hz) [90]. 

##### CNC-Aerogels

CNC are highly crystalline rigid rods with the aspect ratio lower than that of CNF; thus, cellulose concentrations slightly higher than in CNF gels are needed to form a percolated network and self-standing aerogels. Neat CNC aerogels with density from 0.08 to 0.1 g/cm^3^ were obtained in ref. [91]. Interestingly, no shrinkage was observed during aerogel preparation. Specific surface area varied from 250 to 600 m^2^/g. No clear trend as a function of cellulose concentration in the gel (or on aerogel density) was recorded. In this case the stable CNC network was formed via hydrogen bonding between hydroxyl groups [91]. Another option to stabilise CNC aerogels’ shape is to use chemical cross-linking. For example, Yang and Cranston produced aerogels based on hydrazone crosslinking and aldehyde-functionalized CNCs [92]. Density varied from 5 to 20 mg/mL and specific surface area was around 250 m^2^/g. These aerogels showed shape recovery under compression and absorbed significant amounts of water (160 g/g) and dodecane (72 g/g) with cyclic absorption capacity. Other polymers may act as a binder of CNC [93]; such approach was often used when gels are freeze-dried.

##### BC-Aerogels

The high purity of bacterial cellulose is especially attractive for biomedical applications [94]. The BC-based aerogels can be produced at very low solid contents resulting in a density as low as 8–9 mg/cm^3^; specific surface area was around 200–250 m^2^/g [95,96]. Shrinking of bacterial cellulose gels during the preparation of aerogels is negligible, most probably because of high “strength” of nanofibrils due to cellulose high molecular weight and crystallinity. BC aerogels were demonstrated to be carriers of dexpanthenol and of L-ascorbic acid for controlled release applications [96]. Surface-modified (oxidized/silanized) and freeze-dried BC were shown to be promising for the selective adsorption of organic solvents and oils [97]. 

##### Nanocellulose Aerogels Application Perspectives

The properties exhibited by nanocellulose-based porous materials (freeze-dried or with supercritical CO_2_) suggest using them in numerous applications as absorbent or absorbents in bioremediation, as insulating materials, as carbon precursors, in electrical devices and for energy storage, in food packaging and scaffolds for biomedical application [34,98,99,100,101,102,103]. 

The use of nanocellulose-based porous materials in biomedical and pharmaceutical sectors has been focused on two main applications: 3D cell culture scaffolds and drug delivery. The interconnected porous structure exhibited by bacterial cellulose favours the cellular infiltration and allows the nutrient and waste exchanges. The cell culture in nanocelluloses-based aerogels results in a cell death less than 5% after 72 h of cell growth, allowing its effective application as three-dimensional support for cell growth [104]. For example, Liu et al. studied the cell functions on CNF-based porous materials using epithelial-derived and hematopoietic-derived cells obtaining outstanding results for cell growth, survival, and proliferation, making these materials good candidates for tissue engineering applications [104]. Nanocellulose was also reported to be promising as drug delivery system [105]. For example, Valo et al. used CNF-based aerogels from different cellulose sources for the immobilization of beclomethasone dipropionate nanoparticles for oral drug delivery systems [106]. This research concludes that drug release can be controlled by the modulation of the matrix properties resulting in very different release profiles [106]. The mucoadhesive properties and the floating ability of the nanocellulose-based aerogels are responsible for the stability of these materials in oil–water and air–water interfaces, increasing drug bioavailability compared to the intravenous and oral application of the pure drug solution [107]. 

Nanocellulose-based aerogels can also be used in electrical devices and energy storage applications. In recent years, supercapacitors have raised great interest as energy storage devices due to their longer cycle life, higher power densities and shorter charging/discharging time compared to conventional batteries. Highly porous materials with mechanical strength and a large surface area, such as nanocellulose-based aerogels, are good candidates for the adsorption of the electrolyte ions for the production of supercapacitors. Zhang et al. prepared a flexible solid-state CNF-based aerogel supercapacitor using silver and polyaniline nanoparticles on the nanofiber surface, observing a relatively high capacitance (176 mF·cm^2^ at 10 mV·s^−1^) [108]. Yang et al. prepared CNC-based aerogels as substrate to produce 3D structures for supercapacitor application. In this case, several conductive materials such as polypyrrole, carbon nanotubes and manganese dioxide nanoparticles were used for increasing the electrical conductivity. The resultant materials were lightweight with excellent mechanical properties, remaining intact when they were compressed in air or in aqueous electrolyte; they also showed shape recovery behaviour, making them a promising material for energy applications [109]. 

Carbonization of nanocellulose-based aerogels is an alternative to the addition of conductive polymers. Carbon aerogels present high specific surface area, high porosity, large number of oxidative groups, high conductivity, stability, and versatility for their applications as supercapacitors. Compared to other carbon templates based on polyacrylonitrile or polybenzimidazole, the nanocellulose-based aerogels exhibit a better performance because of their small pore size, unique porous structure, and are also attractive because of renewability [110]. 

Low density and pore size below few hundreds of nanometers, close to mesopores, make nanocellulose aerogels attractive for thermal insulation, showing even better properties than currently used materials such as polyurethane and polystyrene foams [111]. Some nanocellulose aerogels are classified as thermal superinsulation material due to a thermal conductivity below that of the air (0.025 W/m·K) [34,35]. To tune thermal conductivity, nanocellulose-based aerogels can be functionalized and combined with additives such as nanozeolites, graphene oxide or sepiolite nanorods. Bendahou et al. reported that adding nanozeolites to cellulose nanofibers results in the reduction in the thermal conductivity (18 mW·m^−1^·K^−1^ with 10 wt% nanozeolites) [39]. Wicklein et al. studied the thermal insulation properties of CNF-based aerogel in combination with graphene oxide (GO), together with sepiolite nanorods (SEP) [112]. CNF-GO-SEP hybrid aerogels showed very low thermal conductivity of 15 mW·m^−1^·K^−1^. Methyltrimethoxysilane aerogels were reinforced with silylated CNF network resulting in thermal conductivity around 0.017 W/m·K [113].

The absorption of contaminants in water by high specific surface CNF aerogels is an innovative method for environmental remediation. He et al. studied the use of cationized-CNF aerogel for the removal of Cr (VI) from water [114]. The CNF surface was functionalised with a quaternary ammonium (2,3-epoxypropylmetrhylammoniumchloride). The cationized CNF-based aerogel showed a removal capacity of 99% for Cr (VI) in a 50 min treatment. In addition, these aerogels exhibited only a 5% decrease in their adsorption capacity after several cycles. Sajab et al. produced aerogels from graphene oxide (GO) and CNF and demonstrated a fast methylene blue (MB) adsorption compared to neat CNF-based aerogels [115]. In addition, the functionalization of the active surface sites of GO with Fe(III) allowed the removal of MB by the Fenton adsorption oxidation process. This process offered an advantage for the regeneration of the adsorbent aerogel: the addition of Fe(III) enabled the regeneration of the aerogel for five-cycles with a maximum elimination capacity up to 120 mg/g [115]. The possibility of cellulose surface hydrophobization allows using their aerogels for the removal of organic compounds from contaminated water. 

##### Cellulose II Aerogels (via Dissolution-Coagulation Route)

To obtain cellulose II aerogels, two main ways of cellulose dissolution should be considered, either via cellulose derivatization followed by regeneration (for example, viscose process) or in direct solvents followed by coagulation or precipitation (sometimes also called “regeneration”). Very few articles describe cellulose aerogels made via derivatization-regeneration route. Ookuna et al. reported on cellulose aerogels obtained from viscose process [116]. Specific surface area varied from 15 to 400 m^2^/g, and it was suggested to be used as ion exchangers. The reason of the absence of research on cellulose aerogels from viscose is that cellulose derivatization is performed in concentrated alkali with carbon disulfide followed by ejection of sulfur gases during regeneration; to control this process on laboratory scale is not an easy task. Another example of cellulose aerogels via derivatization-regeneration route is via synthesis of cellulose carbamate [117]. The latter is synthesized by kneading cellulose in the excess of urea at 130 °C followed by the dissolution in NaOH/water. After regeneration, solvent exchange and drying with supercritical CO_2_ cellulose aerogel density was from 0.06 to 0.22 g/cm^3^ and specific surface area around 400 m^2^/g [117].

The most popular way of making cellulose II aerogels is via direct dissolution, similar to other polysaccharides. Because of strong inter- and intramolecular bonds between cellulose macromolecules, it is soluble in a limited amount of specific solvents [65]: based on amine oxides such as N-methyl morpholine-N-oxide monohydrate (NMMO) used for industrial spinning of fibers (Lyocell process), transition metal and amine or ammonium components, aqueous alkali (mainly 6–10 wt% NaOH-water based), ionic liquids, inorganic molten salt hydrates, and Li salts such as LiCl/N,N-dimethylacetamide, which is the most popular solvent used for cellulose characterization and derivatization on laboratory scale. Only some of these solvents are used to make aerogels [62]. 

It is interesting to note that opposite to the majority of bio-aerogels, gelation step is skipped in the preparation of cellulose II aerogels. The reason is that cellulose solutions are not “easy-gelling”, as, for example, alginate or pectin solutions that undergo gelation in the presence of metal ions or due to the change of solution pH. Without gelation, the structure of cellulose II aerogels is formed during non-solvent induced phase separation, when cellulose solvent is washed out by a non-solvent, usually water or ethanol. Within the common cellulose solvents, gelation of cellulose solutions occurs in 6–10 wt% NaOH-water, with time and temperature increase. Often gelation is delayed due to the addition of urea or thiourea or ZnO [118]. 

**Figure 7:**
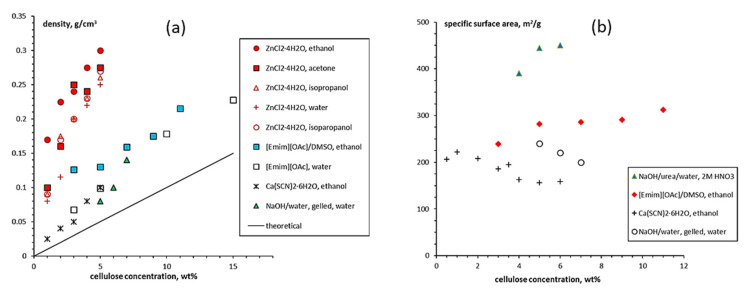
(**a**) Density (data are from the references: [40,121,122,123,124]) and (**b**) specific surface area (data from references [40,122,125,126]) of cellulose II aerogels as a function of cellulose concentration in solution, all for the dissolved microcrystalline cellulose or dissolving pulp in different solvents (first position in the legend) and coagulated in different non-solvents (second position in the legend); the solid line in (**a**) corresponds to the case of no shrinkage.

The density of cellulose aerogels was shown to depend on several factors, one being the concentration of cellulose in the initial solution. It is now well known that shrinkage occurs during all processing steps, and it was demonstrated that higher cellulose concentration leads to lower shrinkage [119,120,121,122]. Because of volume shrinkage, the density of aerogels is always higher than theoretically predicted in the case of no volume change (Figure 7a). Other factors than cellulose concentration may influence aerogel density which still remains to be explored: molecular weight [89], type of solvent, potentially the type of non-solvent, the way how solvent → non-solvent exchange was performed and supercritical drying conditions. Figure 7a shows the data from literature corresponding only to a low molecular weight cellulose. 

**Figure 8:**
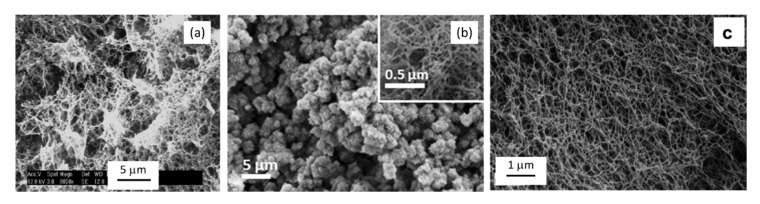
Morphology of cellulose II aerogels made via (**a**) dissolution-gelation in NaOH-water [40] (Adapted with permission from Gavillon, R.; Budtova, T. Aerocellulose: New Highly Porous Cellulose Prepared from Cellulose—NaOH Aqueous Solutions. Biomacromolecules **2008**, *9*, 269–277. Copyright (2008) American Chemical Society), (**b**) dissolution in 1-ethyl-3-methylimidazolium acetate /DMSO [122] (Reprinted by permission from [Springer] [Cellulose] [Cellulose Aero-, Cryo-and Xerogels: Towards Understanding of Morphology Control, Buchtova, N.; Budtova, T., 23, 2585–2595] COPYRIGHT 2016) and (**c**) dissolution in molten Ca(SCN)_2_·4H_2_O [126] (Adapted from The Journal of Supercritical Fluids, 106, Karadagli, I.; Schulz, B.; Schestakow, M.; Milow, B.; Gries, T.; Ratke, L., Production of porous cellulose aerogel fibers by an extrusion process, 105–114, Copyright 2015, with permission from Elsevier).

Specific surface area of cellulose II aerogels is shown in Figure 7b as a function of cellulose concentration in the starting solution. In order to exclude the influence of molecular weight, aerogels based only on dissolved microcrystalline cellulose or dissolving pulp of low molecular weight are shown. The surface area varies from 150 to 450 m^2^/g, but the trends are not clear. It was suggested that the increase of specific surface area with the increase of cellulose concentration is due to the “division” of pores into smaller ones [122]. Somehow a similar trend, but as a function of aerogel density (which is proportional to cellulose concentration as shown in Figure 7a), was reported for the case when cellulose was dissolved in ZnCl_2_·4H_2_O [121]. The morphology of cellulose aerogels obtained via dissolution in different solvents is shown in Figure 8.

The mechanical properties of cellulose II aerogels have been tested under the uniaxial compression. The samples do not buckle, its height uniformly decreases until 60–80% strain; aerogels do not break. In the early publications it was suggested that Poisson ratio is zero [121,123,124], however, recently it was demonstrated that at low densities (below 0.1 g/cm^3^) Poisson ratio is around 0.15–0.2, and it decreases with density increase [89]. Compressive modulus varies from 1 to 100 MPa depending on aerogel density [62]. Usually, the compressive modulus of aerogels is approximated by a power law function of density. Because of the numerous parameters that can influence cellulose II aerogel properties, the exponent in this trend varies from 2 to 4. One of aerogel characteristics that is important and may influence mechanical properties is cellulose crystallinity; unfortunately, it is almost never considered making the comparison of results from different publications difficult. 

Opposite to bio-aerogels based on nanocellulose [34,35,46] or pectin [16,33], cellulose II aerogels are not thermal super-insulating materials. Thermal conductivity was never reported to be lower than 0.025 W/m·K. The reason is most probably too many large macropores and/or thick pore walls. Cross-linking with epichlorohydrin did not help solving this problem [125]. The only way which resulted in cellulose II based thermal super-insulating aerogels was synthesis of interpenetrated cellulose-silica network with both components being hydrophobized [126]. Silica aerogel “inside” the pores of cellulose network decreased the conductivity of the gas phase. Specific surface area of composite aerogel increased from 250–330 m^2^/g for cellulose to 610–750 m^2^/g for composite, and thermal conductivity was 0.021–0.022 W/m·K [126]. 

##### Cellulose II Aerogels Application Perspectives

Bio-aerogels are promising materials for bio-medical applications if no toxic compound is used for cross-linking and the traces of solvents are accepted by regulations. While nanocellulose aerogels and foams have been widely studied as drug delivery matrices or for cells proliferation (see, for example, [127]), very few works tested bio-medical applications of cellulose II aerogels. Biocompatibility was demonstrated for dual-porous cellulose II aerogels made via cellulose dissolution in ionic liquid 1-ethyl-3-methylimidazolium acetate or in calcium thiocyanate; lower cell viability was observed for the case of ionic liquid [128] most probably because of the traces of the solvent. Aerogels based on phosphorylated cellulose showed good hemocompatibility [129]. Cellulose II aerogels made by using only non-toxic cellulose solvents can be considered for bio-medical applications. 

Porous materials are widely used for adsorption and absorption of pollutants such as oil, organic solvents, dyes, and heavy metals. The advantage of aerogels is their high performance and disadvantage is high price. In addition to high performance, multiple reuse and easy degradation of the absorbent or adsorbent need to be considered. As far as cellulose aerogels are concerned, functionalization and/or pyrolysis is needed to increase the efficiency and selectivity. This topic is discussed in more details in Section 3.1.3 as multiple approaches are known. 

Carbon aerogels from pyrolyzed synthetic polymer aerogels are suggested to be used in energy and electrochemical application: for hydrogen storage, in supercapacitors, lithium-ion batteries and as catalyst supports. Very few is known on carbons from pyrolyzed cellulose II aerogels. Literature reports on pyrolyzed freeze-dried dissolved-coagulated cellulose: for example, when NaOH/water was used as cellulose solvent, supercapacitor electrodes were prepared when nitrogen-doped [130], KOH activated [131], and CO_2_ activated [100,132]. When MnO_x_/N doped, the same carbons were tested as monolithic catalysts [133]. Carbons from pyrolyzed cellulose II aerogels (cellulose was dissolved in NaOH/water and gelled) were demonstrated to be very promising materials as cathodes in Li/SOCl_2_ primary batteries [134]. The remarkable fact is that despite a severe shrinkage, those carbons kept the shape of their aerogel precursor. “Green” electrocatalyst was prepared from the same carbon aerogels doped with platinum [134,135]. These promising results show numerous prospects in using cellulose and, potentially, lignocellulose matter, for high end applications.

#### 3.1.2. Lignin Aerogels

**Figure 9:**
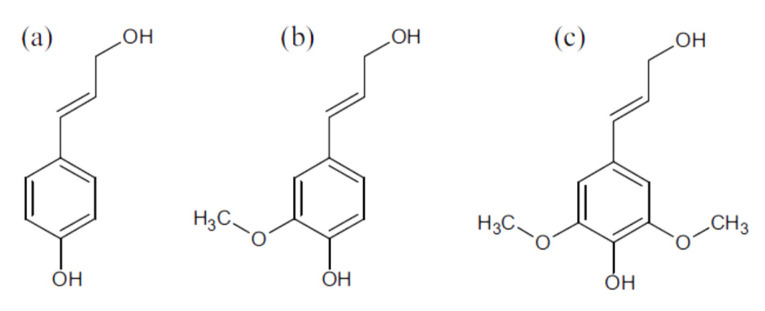
Structural units of lignin: (**a**) p-coumaryl alcohol (4-hydroxyl phenyl), (**b**) coniferyl alcohol (guaiacyl), (**c**) sinapyl alcohol (syringyl) [141].

Lignin is the most abundant natural aromatic polymer, which comprises 20–30% of woody plant cell walls [136,137]. The lignin heteropolymer is an integral cell wall constituent that significantly influences the physical properties of plants via its involvement in architectural support, water transport, and defense [137,138]. Lignin is developed by oxidative coupling of three major C6-C3 (phenylpropane) monomers. The phenolic polymer of lignin comprises guaiacyl alcohol, syringyl alcohol and ρ-hydroxyl alcohol (c) (Figure 9) [139,140]. Lignin density is around 1.3–1.4 g/cm^3^ and refractive index is 1.6. The combustion energy of lignin used for energy recovery is 29.5 MJ/kg [139,140]. 

Lignin is biosynthesized via a plant-peroxidase-catalyzed oxidation of (methoxy-)substituted para-hydroxycinnamyl alcohols [137]. The benzylic hydroxy groups are introduced via quinone methide intermediates [137,142]. A critical review on the methods of lignin isolation was recently published by Bhat et al. [137]. The commonly used method to isolate lignin from wood is a thorough milling of the plant material, followed by extraction with dioxane/water; the extracted material is referred to as milled wood lignin. The yields are usually low, and the possibility of chemical changes occurring during the isolation process must always be taken into account [143]. Methods for lignin separation from plants are alkali process, sulphite process, organic solvent extraction, and ball milling [144]. Among them, only alkali and sulphite process are currently the main sources of commercial lignin. 

Lignin can be used as dispersing, binding, complexing, and emulsion-stabilizing compound [145]. The most common use of lignin is as an additive. Lignin is also used as an additive to animal feed to improve pellet quality and production efficiency due to its excellent bonding property. Lignin can be an additive to crude oil when drilling muds, it is a raw material for the synthesis of vanillin or non-ionic surfactants when pyrolyzed for oil recovery [145]. A promising added-value product from lignin is biodiesel [146]. Overall, the isolation and utilization of lignin as one of the major constituents of lignocellulosic biomass is considered for the second- and third-generation biorefinery approaches. While till now lignin have been mainly used for energy generation, there is broad consent that its controlled depolymerization to fine chemicals or its processing to innovative materials is a future-oriented measure to improve the overall performance of respective biomass processing units [147]. 

The majority of publications report using lignin (L) as one of the components of aerogels, often mixed with phenol (P) and formaldehyde (F) [53], or with tannin and formaldehyde [141], or with resorcinol (R) and formaldehyde (F) [148]. For example, freeze-dried and supercritically dried porous materials were made from different LPF compositions [53]. The bulk density of these aerogels varied from 0.19 to 0.38 g/cm^3^ and density was inversely proportional to lignin and formaldehyde content. Pore-size distributions were found to mainly depend on the composition, but not on the drying mode. These aerogels revealed higher amount of macropores and less mesopores. The thermal conductivity of aerogels with 80% lignin was lower than that of 50% lignin, 0.041 vs. 0.045 W/m K, respectively. The compressive modulus of aerogels decreased with increasing amounts of lignin from 5.89 and 22.86 MPa for 80% and 50% lignin content, respectively [53]. 

When aerogels were synthesised from (tannin+lignin)/formaldehyde in different proportions, volume shrinkage was from 26% to 39%, and bulk density from 0.28 to 0.39 cm^3^/g and porosity from 72% to 87% [141]. The skeletal density measured by helium pycnometry was 1.44 ± 0.03 g/cm^3^ and BET specific surface was 170–440 m^2^/g. Thermal conductivities were not very low, from 0.041 to 0.045 W/m·K. Compression tests evidenced brittle fracture. 

**Figure 10:**
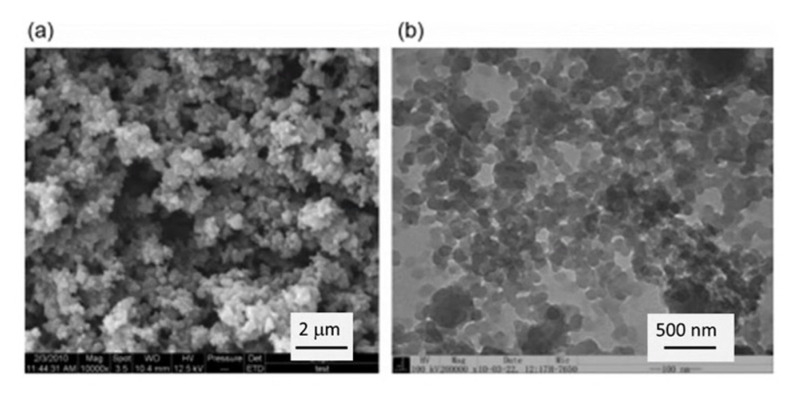
SEM (**a**) and TEM (**b**) images of lignin-resorcinol-formaldehyde aerogel with 50% lignin, adapted from [148].

In LRF aerogels most of the pores are about 50 nm wide (Figure 10), as is typical for mesoporous materials, and specific surface area was from 191 to 552 m^2^/g. It increased with decreasing lignin concentration [148]. Mesopore volumes were from 0.834 to 0.895 cm^3^/g. The bulk density of LRF aerogels were tuned by the total matter concentration, lignin concentration and molar ratio LR/F.

Ultralight and fire-resistant lignin-based aerogel was obtained by hydrothermal treatment in the presence of graphene oxide (G) [149] and freeze-drying. LG aerogel exhibits an interconnected 3D framework with pores ranging from nanometers to micrometers. The density was from 3.0 to 8.2 mg/cm^3^ and specific surface area 270 m^2^/g. LG aerogel did not break under compression and showed high elastic recovery. The LG also showed excellent fire-resistant property. Due to high porosity, hydrophobic nature and high elastic recovery LG aerogels were shown to be efficient in oil and organic solvents separation.

Lignin extracted from wheat straw was cross-linked with oligo(alkylene glycol)-α,ω-diglycidyl ethers and dried with supercritical CO_2_ [147]. The average skeletal density of the obtained materials was 1.07 g/cm^3^ independent on the type of crosslinker used, bulk density varied from 0.2 to 0.4 g/cm^3^. Based on these values the porosity of the lignin aerogels was calculated, it varied from 75% to 88%. The obtained aerogels had a surface area of up to 120 m^2^/g and the thermal conductivity was rather high, around 0.05 W/m K.

Lignin can also be mixed with polysaccharides to make aerogels. For example, lignin-alginate aerogel with bulk density 0.03–0.07 g/cm^3^, surface area up to 564 m^2^/g and pore volume up to 7.2 cm^3^/g was synthesized [150]. The linear shrinkage caused by solvent exchange and supercritical drying was in the range of 20–35%. 

Till now the majority of studies on using lignin for making aerogels concern the exploration of their properties. Karaaslan et al. [151] argued that, despite the limited number of studies on lignin-based aerogels, it has been shown that lignin is a promising precursor that could be a substitute for synthetic and toxic raw materials, such as resorcinol and phenol, in making organic and carbon aerogels. Some of the potential products that have been suggested for lignin-based aerogels so far are thermal insulators and carbon electrodes for energy storage devices such as supercapacitors. More research on lignin-based aerogels should be performed in order to better understand the structure–property relationships and explore other possible aerogel products derived from lignin.

#### 3.1.3. Hemicelluloses Aerogels

Hemicelluloses are one of the main components of plant raw materials and have high economic potential for bio-based products due to their attractive advantages such as: renewability, biodegradability, biocompatibility, abundant resources, and non-toxic properties. Hemicelluloses are found as organic wastes or byproducts of forest and agricultural products. Depending on the origin, the composition of hemicelluloses varies. The main source of hemicelluloses is wood, straw, corn cobs or sugarcane bagasse [152]. Lignocellulosic biomass contains about 20–30% of hemicelluloses [153]. 

A hemicellulose (polyose) is a heterogeneous group of simple sugars and their derivatives, stabilized by β-1-4 glycosidic bonds, less frequently by β-1-3 glycosidic bonds, which form branched chains [154]. In contrast to cellulose, which is based on the anhydroglucose repeated unit, hemicelluloses contain different sugars and is of low molecular weight. The major hemicellulose components are:

- Pentoses (C_5_H_8_O_4_)_n_ (l-arabinose, d-xylose)
- Hexoses (C_6_H_10_O_5_)_n_ (d-galactose, d-glucose, d-mannose)
- Uronic acids (d-glucuronic acid, d-galacturonic acid)

**Figure 11:**
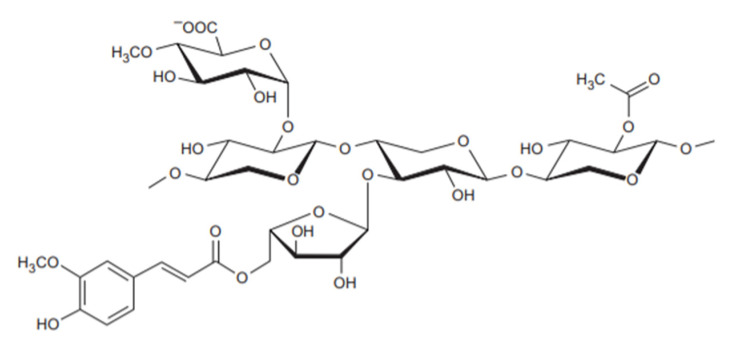
Structure of hemicellulose [156] (Reprinted from Water Extraction of Bioactive Compounds, From Plants to Drug Development, Siti Machmudah, Wahyudiono, Hideki Kanda, Motonobu Goto, Chapter 3—Hydrolysis of Biopolymers in Near-Critical and Subcritical Water, 69–107, Copyright 2017, with permission from Elsevier).

Sugars such as l-rhamnose and l-fructose are present in much smaller amounts [155]. Figure 11 shows the typical structure of hemicellulose, containing linear and highly branched chains of sugars with covalent and hydrogen bonds [156]. 

The composition of hemicelluloses depends on the source and pre-treatments. Xylan is a main component of hemicelluloses and is abundant in softwood pulp. Xylan is a water-binding polysaccharide, and its presence plays an important role in the rheology and drainage of gels obtained from birch pulp, traditionally bleached by sequential method.

Hemicelluloses are less resistant to dilute acids and, unlike celluloses, they dissolve in dilute alkalis. Many different methods can be used to extract hemicellulose from woody and non-woody biomass [157,158] such as: alkaline extraction being the most popular [153,159], ionic liquid extraction [160], hot water extraction [161], alkaline hydrogen peroxide extraction [162], microwave assisted extraction [163], steam treatment [164], and acid pre-extraction [165]. 

Hemicelluloses are used in various domains such as foods and feeds [166], medical and pharmaceutical application [167], in cosmetics [168] or as coatings for packaging and paper [169]. Chemical modification can lead to properties such as conductivity, thermoplasticity, or hydrophobicity, which significantly increases the application possibilities of hemicelluloses [170]. Composites with hemicellulose can be used in the form of hydrogels, adsorbents, coatings, sensors or as drug carriers [171]. Recently, hemicellulose started to be considered as an additive for 3D printing of functional bioproducts, for example, when mixed with PLA [172]. Hemicellulose can also be used as a carbon source for the synthesis of porous activated carbon for high-performance supercapacitors [173].

Various hemicelluloses have been used for the preparation of porous materials [20], but neat hemicellulose aerogels are very rare, most probably because of their weak mechanical properties not allowing the fabrication of “self-standing” samples. Hemicelluloses such as xylans, β-glucan, xyloglucan and arabinoxylan were used, in most cases there were mixed with other polymers. 

**Figure 12:**
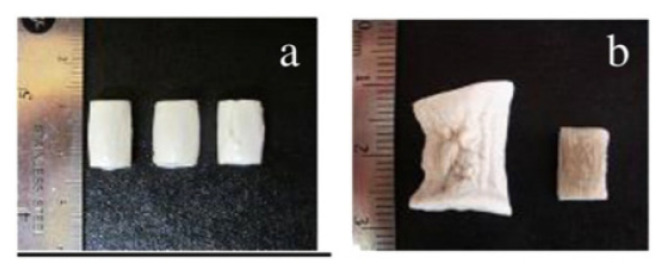
β-Glucan samples: (**a**) aerogels based on 5, 6 and 7% solution (from right to left) and (**b**) 5% freeze dried (left) and 5% air dried samples [174]. Reprinted from Food Res. Int., 48, Comin, L.M.; Temelli, F.; Saldaña, M.D., Barley Beta-Glucan Aerogels Via Supercritical CO_2_ Drying, 442–448, Copyright 2012, with permission from Elsevier.

Barley β-glucan solutions were used to make gels and the influence of drying (freeze-drying, with supercritical CO_2_ and in ambient conditions) on the properties of the material was studied (Figure 12) [174,175]. Air-dried samples had the highest density (0.67 g/cm^3^), as expected, as compared to that from freeze dried (0.17 g/cm^3^) or supercritical CO_2_ dried (0.20 g/cm^3^) gels. Specific surface area of aerogels was within 160–170 m^2^/g. 

Arabinoxylan was extracted from wheat flower and aerogels were prepared from supercritically dried gels [176]. Higher volume shrinkage, up to 80%, was recorded during aerogel preparation. Density was not reported, specific surface area was rather low, around 50 m^2^/g. 

Porous materials based on arabinoxylan containing lignin and reinforced with CNF were prepared via cross-linking with citric acid and freeze-drying [177]. The density was very low, about 0.02 g/cm^3^. The presence of CNF increased the mechanical properties of the samples under compression. The authors reported high water absorption, up to 70 times the weight of the dry sample. A somewhat similar approach was taken in ref. [178,179]: xyloglucan or xylan was reinforced by CNC and dried using unidirectional and isotropic freeze-drying. A higher concentration of cellulose resulted in higher compressive modulus and higher shape stability when immersed in water [177]. In both publications, specific surface area was not reported, but is supposed to be very low as pore dimensions were of several tens of microns.

#### 3.1.4. Aerogels from Entire Lignocellulose

Surprisingly, not much is known about aerogels based on entire lignocellulose. One of the problems is to find a common solvent for cellulose, lignin, and hemicellulose. For example, aerogels from biomass were obtained by dissolving bagasse in LiCl/DMAc and freeze-dried from tert-butanol [180]. The density of aerogels was from 0.088 to 0.236 g/cm^3^ and specific surface area was 185 m^2^. The analysis of FTIR spectra revealed that a small amount of lignin and hemicellulose was washed out during the preparation.

Ionic liquids have been shown to dissolve wood and can thus be used for making lignocellulose aerogels. Aaltonen et al. reported on aerogels prepared from spruce wood and compared their properties with aerogels made from the mixtures “imitating” wood: cellulose, lignin (here, soda lignin) and xylan [181]. The solvent was 1-butyl-3-methylimidazolium chloride. Density of aerogels was from 0.025 to 0.114 g/cm^3^ and the specific surface area from 108 to 539 m^2^/g. The highest value of specific surface area was for neat cellulose coagulated in ethanol; lignocellulose-based aerogel was with rather low surface area, around 110–120 m^2^/g. The addition of lignin and xylan to cellulose solution resulted in the increase in density and decrease of surface area as compared to neat cellulose aerogels. A similar approach was used by Sescousse et al.: microcrystalline cellulose and organosolv lignin were dissolved separately in 8%NaOH-water, mixed and aerogels prepared [182]. Gelation of cellulose was accelerated in the presence of lignin. Part of lignin was washed out, depending on pH of the coagulation bath. 

Carbon aerogels were prepared from kraft lignin and TEMPO oxidized nanofibrillated cellulose, both dispersed in water, freeze-dried and pyrolyzed [183]. The porosity of this porous carbon increased from 91.6% to 93.4% with increasing CNF content, while the density decreased from 0.18 to 0.14 g/cm^3^. The specific surface area of the carbon with 12 wt% of CNF in its precursor had a specific surface area of 806 m^2^/g. CO_2_ adsorption capacity was tested: it was 3.39 mmol/g at 273 K and 100 kPa and was further improved to 1101 m^2^/g and 5.23 mmol/g by washing. When assembled as electrodes in a supercapacitor, the carbon reached a specific gravimetric capacitance of 124 F/g at 0.2 A/g and an aerial capacitance of 1.55 F/cm^2^ at 15 mA/cm^2^, overcoming many other types of porous carbon materials reported in the literature.

Some works use pulps (hardwood prehydrolysis kraft pulp, hardwood sulfite pulp, hardwood kraft pulp) which were phosphorylated and dissolved in NMMO to make hemocompatible aerogels [129]. All aerogels showed good hemocompatibility and inflammatory response. Non-phosphorylated counterparts had similar density (around 0.06 g/cm^3^) and specific surface area (around 240–280 m^2^/g). Various pulps, including unbleached ones, were used to make aerogels using NMMO as solvent, but the influence of lignin or hemicellulose was not studied [119].

Birch pulp (92% cellulose, 7 wt% of hemicelluloses and less than 1 wt% of lignin) of various degrees of polymerization of cellulose was dissolved in 8%NaOH-water, with kraft fibers added for the reinforcement [184]. Density varied from 0.1 to 0.2 g/cm^3^ and specific surface area from 150 to 350 m^2^/g. The presence of too many of non-dissolved and thus non-porous fibers decreased the specific surface area of aerogels and compromised material mechanical properties. The results demonstrated that cellulose complete dissolution is not needed for making strong aerogels with high specific surface area using non-toxic low-cost solvent, 8 wt% NaOH-water.

Several publications report on “aerogels” using lignocellulose, but most of them were obtained via freeze-drying and are thus with very large pores and should be called “foams” or “sponges”. In most of them specific surface is not reported. For example, wood was delignified, hemicellulose removed, sample freeze-dried, and the obtained porous material was shown to be promising for solar desalination [185]. Fibers from rice straw were mixed with PVA, freeze-dried and hydrophobized with methyltrimethoxysilane; the materials had a density of 0.05–0.06 g/cm^3^, a thermal conductivity of 0.034–0.036 W/m·K, and an oil adsorption capacity up to 13 g/g [186]. When cotton stalks (21% of Klason lignin) were dissolved in 1-allyl-3-methylimidazolium chloride/dimethyl sulfoxide and freeze-dried from tert-butanol, the density was from 0.11 to 0.15 g/cm^3^ and specific surface area from 30 to 100 m^2^/g depending on biomass concentration [187].

#### 3.1.5. Functionalization as a Tool to Enhance the Properties of Lignocellulose Porous Materials for Environmental Applications

To achieve targeted applications of lignocellulose, surface modification and different treatments are often needed. Various routes for design of functional materials can be achieved and combined to take advantage of inherited properties of lignocellulose-based materials and of material porous structure. Here we consider not only lignocellulose-based aerogels per se, but also efficient cellulose-based macroporous materials.

Widespread use of oil and various organic substances have led to various cases of environmental pollution due to accidents and poor safety measures. For example, the Gulf of Mexico in 2010 had one of the largest ecological accidents that lead to about 4.9 million barrels of crude oil being released in the ocean, and marine ecosystems and wetlands were devastated [188]. There is an urgent need in advanced materials that can effectively recover and separate oil from water to preserve aquatic ecosystems, protect clean water drinking sources and avoid usage of methods as controlled burns [189]. Table 1 summarizes modification routes of functionalized porous cellulose adsorbents for oil-water treatment. Chhajed et al. [190] evaluated the efficacy of CNF/polyvinyl alcohol (PVA) modified with stearic acid chloride as a superabsorbent for treating oil and organic chemical spills in water and demonstrated high selectivity and repeated reusability for at least 15 times, achieved by mechanical squeezing. A different approach for cellulose foam modification was proposed by Li et al. [191] with deposited copper nanoparticles which were reported to be very effective for oil and other organic substance adsorption. Usage of modifier in combination with cross-linking reaction is also proposed in literature to achieve higher mechanical properties [192]. Bidgoli et al. used an esterification reaction followed by cross-linking with hexamethylene diisocyanate (HMDC) to modify cellulose for foam production which resulted in good oil absorption and retention ability but recycling with centrifuge resulted in decrease of sorption capacity by almost half starting from the second cycle [192]. Xu et al. prepared porous cellulose grafted with epoxidized soybean oil (ESO) in mild conditions, but most notably reported crude oil initial absorption capacity of 37 g/g which after 30 adsorption cycles decreased to 33 g/g or above 90% of initial capacity [193]. Aalbers et al. prepared CNC foams functionalized with a methacrylate and then further modified them using thiol-ene click chemistry to prepare five different types of surface modifications, but the authors noted that extensive modification and use of organic solvents lead to the collapse of porous structure and the best result yielded on average 2.90 mL/g per five repeated cycles of xylene absorption [194]. 

Silane based modifiers, such as trimethylchlorosilane (TMCS) [195], hexamethyldisilazane (HMDS) [195], methyltrimethoxysilane (MTMS) [196,197], hexadecyltrimethoxylan (HDTMS) [198] and methyltrichlorosilane (MTCS) [199] have been known as popular cellulose modifiers for various applications. For example, HDTMS-modified cellulose foams absorbed up to 79 g/g of motor oil, 162 of sunflower oil and retained good performance after 20 sorption cycles. MTMS-modified cellulose showed similar results [196,197,198]. In addition to oils, modified cellulose foams showed potential in absorption of various toxic organic solvents, thus extending their application for chemical spill recovery [190,191,192]. 

Atmospheric carbon dioxide (CO_2_) has been rapidly increasing since the industrial revolution from 280 ppm to values above 400 ppm in recent years [200]. This has led to many environmental issues that disrupt climate conditions around the world. As a consequence, research focused on the carbon dioxide capture and conversion [201]. Aerogels are suitable for adsorption process, but cellulose requires surface modification to adsorb CO_2_. Functionalized cellulose aerogel adsorbents for CO_2_ adsorption are summarised in Table 2. CNCs were aminated in the gas phase, and 1.59 mmol/g adsorption capacity was achieved which remained high at about 1.5 mmol/g after five cycles compared to untreated CNC aerogel (0.19 mmol/g) [202]. Moreover, 3-aminopropyltriethoxysilane (APTES) was grafted on porous cellulose resulting in CO_2_ adsorption capacity of 1.20 mmol/g, and it retained excellent stability within 20 adsorption-desorption cycles with dry CO_2_ [203]. Phalimide modified CNF were studied with different loading of amino groups resulting in adsorption values of 5.20 mmol/g [204]. CNF were aminated achieving a high CO_2_ adsorption capacity of 1.78 mmol/g as well as good regeneration reported above 10 cycles [205]. The amine loading of 9.02 mmol/g resulted in the CO_2_ uptake of 1.02 and 0.35 mmol/g at 25 °C and 15 kPa by chemical and physical interactions, respectively [206].

Wastewater treatment to remove dyes is another major environmental issue where lignocellulosic porous materials can be applied [207]. Some examples of using porous cellulose for dyes’ removal are shown in Table 3. Functionalization of cellulose through polydopamine (PDA) coating was reported to be selective for methylene blue adsorption with highest values of 96.70 mg/g [208]. Hexagonal copper (II) sulphide was deposited onto cellulose using a photoinitiated reaction retaining good capacity after 5 reuse cycles with an initial value of 59 mg/g for methylene blue [209]. The silane modified CNF foams showed a high adsorption capacity for crystal violet dye with a value of 150 mg/g [210]. Zeolitic imidazolate framework was used for the preparation of a hybrid metal-organic aerogel based on cellulose for high adsorption (up to 617 mg/g) of methyl orange dye that can retain its adsorption capacity above 90% after five adsorption-desorption cycles [211]. 

Heavy metal pollution has led to severe environmental problems around the world as some industries produce contaminated wastewaters and pollute sewage systems that result, for example, in the spread of water-related diseases [207]. Reusable polyethylenimine (PEI) grafted porous cellulose was able to remove hexavalent chromium from aqueous solutions with an initial capacity of 229.10 mg/g [212]. CNF was combined with PEI via electrostatic interactions to produce aerogel for heavy metal removal from water with the maximum adsorption capacity of 175.44 mg/g Cu(II) and 357.44 mg/g Pb(II), and it was shown to be easily regenerated by ethylenediaminetetraacetic acid (EDTA) [213]. Li et al. prepared cellulose-based carbon aerogel that can adsorb 55.24 mg/g Cu(II) ions [214]. Wang et al. synthesized carboxylated cellulose with magnetic nanoparticles for Cu(II) removal from water and reported the maximum adsorption capacity of 73.70 mg/g at 25 °C [215]. 

Porous cellulose applications are not limited to water purification and CO_2_ removal. Giese et al. reported on mesoporous photonic CNC with magnetic properties for potential application as electromagnetic interference shielding materials [216]. Nemoto et al. prepared TEMPO oxidized CNF foams suitable for application as air filters [86]. Cross-linked CNC/polysilsesquioxane showed high adsorption capacity for both water and toluene [217]. Poly(ionic liquid) was used to modify cellulose aerogels via Schiff base reaction enabling applications like proteomic analysis, medical diagnosis and sensors [218]. Cellulose based catalyst with good reusability was suggested in [219].

The majority of porous cellulose absorbents and adsorbents mentioned above are not aerogels with a high specific surface area. Recent advances in cellulose modifications open up numerous prospective routes for making functionalized lignocellulose aerogels for effective clean ups of oil and of chemical spills. Being renewable and biodegradable, lignocellulosic materials have a huge role in sustainable functional material development and many applications still remain to be discovered.

### 3.2. Aerogels from Marine Polysaccharides

Marine polysaccharide resources are gaining importance as promising and sustainable feedstocks for the production of food, materials, chemicals, and energy that are upwardly growing worldwide. The scope and potentials of seaweeds for the production of diverse bioproducts of commercial importance have convincingly been demonstrated [223,224]. From a biorefinery perspective, a recent study on the sustainable process for valorizing the biomass of marine resources showed a high rate of biomass utilization (93%) with integrated processes combined with a high throughput [225]. This is very encouraging for the development of bio-aerogels from marine biomass.

#### 3.2.1. Aerogels from Alginate

**Figure 13:**
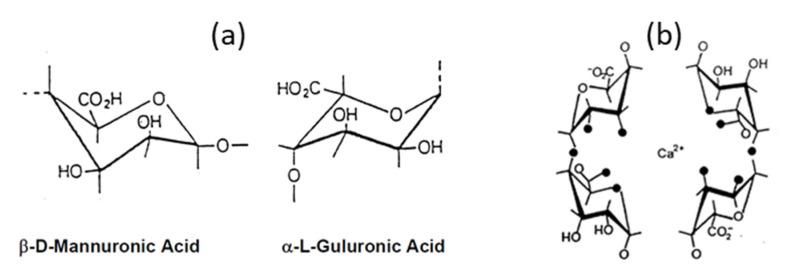
Alginate two main building blocks (**a**) and generalized egg-box model (**b**).

Alginate is an important marine polysaccharide with a chemical structure that consists of 1,4-glycosidically linked α-l-guluronic acid (G) and β-d-mannuronic acid (M) of varying composition and sequence of the different blocks (MM and GG blocks, and MG or GM blocks) (Figure 13a) [226]. Alginate is commercially extracted from brown seaweed or algae (Phaeophyceae), typically including *Laminaria hyperborea*, *Laminaria digitata*, *Laminaria japonica*, *Ascophyllum nodosum*, and *Macrocystis pyrifera* [227]. Generally, the extraction process of alginate consists of three major steps: pre-extraction, neutralization, and precipitation/purification. The idea behind the extraction of alginate from the seaweed is to convert all the alginate salts present in the cell wall to the sodium salt, dissolve this in water, and remove the seaweed residue by filtration. The alginate must then be recovered from the aqueous solution. The recovery after alkaline extraction and separation is carried out by adding acid to the sodium alginate solution. The extract is then filtered, and sodium carbonate is added in order to precipitate alginate in the form of a salt. After additional purification steps, water-soluble sodium alginate is produced with a typical yield between 12% and 45% on a dry weight basis, depending on what type of brown seaweed was used for the extraction. Alginates extracted from different sources further vary in their M and G contents as well as the length of each block, and more than 200 different alginates are currently being manufactured. Owing to its favorable properties, such as biocompatibility and ease of gelation, alginates are widely used in dentistry, wound dressing materials, and as a stabilizer and thickener in food [228].

Alginate is in particular recognized for its ease of gelation in the presence of divalent or trivalent cations which helps the preparation of aerogels [227]. One example of conventional ionic gelation of alginate is with divalent cations, typically Ca^2+^ that follows the “egg-box” model (Figure 13b) with gel structures dependent on the gelation kinetics, which can be tuned by the controlled release of the cation, the temperature, and the cation nature. Furthermore, the alginate source, M-G ratio and the molecular weight of the polysaccharide influence the gelation, and in turn lead to different physicochemical and mechanical properties of the resulting gels and the subsequent aerogels [227]. 

The structure and properties of alginate aerogels in the form of particles have been reported and their production has been optimized through various continuous approaches. Baudron et al. [229] produced microparticles in rather large quantities using a continuous approach of the emulsion-gelation method. The size of aerogel particles was shown to be directly correlated with emulsion droplets; aerogels displayed a specific surface area of 545 ± 30 m^2^/g. Şahin et al. [230] reported on the influence of different ions on the aerogel properties. The lowest density and highest specific surface area were observed for Cu^2+^ (0.043 g/cm^3^ and 573 m^2^/g, respectively), followed by Ca^2+^ (0.056 g/cm^3^; 450 m^2^/g), and Zn^2+^ (0.065 g/cm^3^; 236 m^2^/g).

**Figure 14:**
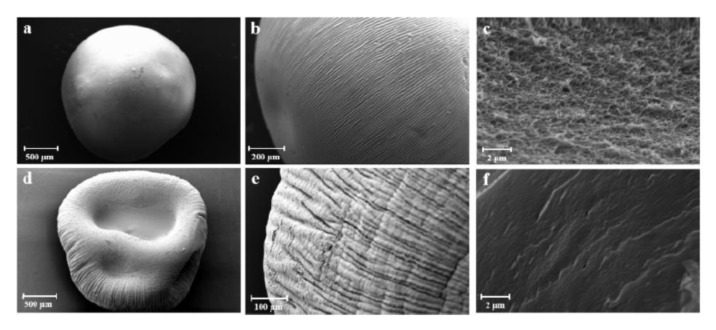
SEM images of: (**a**) alginate aerogel spherical beads after scCO_2_ drying; (**b**) magnification of surface; (**c**) and internal porous network; (**d**) collapsed structure of alginate bead after freeze-drying; (**e**) magnification of surface; (**f**) and internal structure. Reprinted from [232].

Alginate aerogel beads obtained after ionic gelation using dripping [231]/prilling technique [232], or CO_2_-induced gelation [59], presented specific surface areas from 271 to 537 m^2^/g, depending on the physicochemical properties of the alginate source, its gelation and drying approach. In contrast, freeze-dried beads displayed lower specific surface areas between 0.8 and 246 m^2^/g. Figure 14 shows examples of the appearance and structure of alginate aerogel beads after scCO_2_ and freeze drying. The shrinkage of alginate aerogels mainly takes place during the solvent-exchange performed prior to the supercritical drying. Shrinkage from 6% to 70%, or even expansion from 17% to 75% due to swelling have been reported depending on the molecular weight of the alginate and the solvent used. The molecular weight of alginate has further been shown to affect the stability and properties of aerogels upon storage, where medium molecular weight alginate was especially stable after three months of storage at 25 °C and 65% of relative humidity [232].

The water absorption capacity of alginate after ionic gelation was reported in the range of 7 g/g after freeze drying [233] and up to 20 g/g after scCO_2_ drying [18]. For saline (0.9 wt% aqueous NaCl solution) alginate aerogels (specific surface area 549 m^2^/g) absorbed as much as 120 g/g [18].

The development of hybrid alginate-based aerogels, in which other components have been combined with alginate to add or improve functionality has been a frequently applied strategy to achieve a multifunctional aerogel. Aerogel microcapsules, with an antibiotic loaded pectin core, and an alginate shell were prepared by a prilling technique using a coaxial nozzle for drop production in CaCl_2_ solution followed by supercritical drying [234]. The ability of the alginate/pectin core-shell aerogels to swell in contact with wound fluids and the drug release rate was reported to directly correlate with the concentration of alginate. The developed aerogels find potential application in the treatment of both acute and chronic infected wounds. 

Freeze dried alginate was mechanically reinforced by more than 670% by incorporation of clay and pH adjusted gelation, from a compressive modulus of 6.6 MPa to 51 MPa, and the flame-retardant behavior was further improved in these composite porous materials [235]. Another study reported on the enhancement of the mechanical properties of freeze-dried alginate by almost 2900% through incorporation of graphene oxide into polyacrylamide, and combining ionic crosslinking of alginate with covalent crosslinking of polyacrylamide to form a strong hybrid double network [236]. In addition, the graphene oxide significantly increased the catalytic activity, but the specific surface area of the freeze-dried alginate was very low (15 m^2^/g).

The use of alginate aerogels as precursors for carbon aerogel has been another approach to target specific properties and structure. An alginate/chitosan aerogel showed a specific surface area of 399 m^2^/g after ionic crosslinking and supercritical drying, and this was further increased to 549 m^2^/g after carbonization [237]. Carbon aerogels from freeze-dried alginate displayed a specific surface area between 157 to 230 m^2^/g, depending on the ionic gelation conditions and carbonization temperature [238,239], and Cu-doped aerogels further displayed excellent electrochemical performance (414.4 F g^−1^; scan rate: 0.3 mV s^−1^). The multifunctional behavior of the Cu-doped carbon aerogels from alginate was reported to have great potential as supercapacitor in the energy storage field.

The development of alginate aerogels provides several opportunities to adjust their structure and properties through gelation and drying conditions. In addition, their biocompatibility, biodegradability, inherent low flammability, and high absorption ability can be combined to achieve multifunctional aerogels with excellent properties that makes them suitable for a wide range of biomedical and environmental applications such as regenerative medicine (drug delivery, tissue engineering), absorbent, insulation, and catalysis applications [31]. Examples of recently reported applications of alginate aerogels are reviewed in the following section. 

For biomedical applications such as tissue engineering, alginate aerogels are attractive owing to their hydrophilic nature and structural similarities to human tissues [227]. Raman et al. [13] reported on the preparation of alginate aerogels enriched with calcium, zinc, and silver cations for advanced wound care with high cell and tissue tolerability and controlled compound release rates during wound healing. The cation-enriched alginate aerogel absorbed more than 60 times its own weight in water. In particular, the swelling of Zn-enriched alginate in a 4-(2-hydroxyethyl)-1-piperazineethanesulfonic acid buffer led to quantitative metal transfer into supernatants, which indicated effective anti-inflammatory activity. Batista et al. [240] exploited the capacity of alginate to maintain a moist environment together with the antimicrobial activity of chitosan to prepare hybrid alginate-chitosan aerogels for wound healing applications via emulsion-gelation method. The resulting aerogel displayed cytocompatibility and antibacterial activity, with a specific surface area of 162–302 m^2^/g.

**Figure 15:**
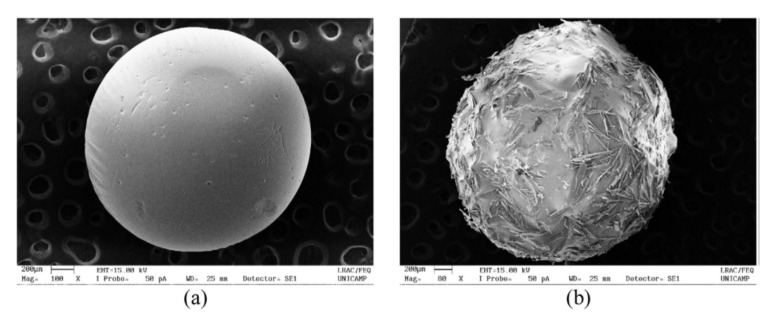
SEM surface morphology of alginate particle: (**a**) after scCO_2_ drying and; (**b**) after resveratrol loading [242]. Reprinted from The Journal of Supercritical Fluids, 163, dos Santos, P.; Viganó, J.; de Figueiredo Furtado, G.; Cunha, R. L.; Hubinger, M. D.; Rezende, C. A.; Martínez, J., Production of resveratrol loaded alginate aerogel: Characterization, mathematical modeling, and study of impregnation, 104882, Copyright 2020, with permission from Elsevier.

The selection of a suitable polymeric carrier is crucial for the preparation of drug delivery systems. In this context, the use of alginate is promising owing to its biodegradability, biocompatibility, and non-toxicity. A common production approach of alginate aerogels for drug delivery systems has been the formation of beads upon gelation in combination with scCO_2_ drying, resulting in mean particle diameters ranging from about 2 to 2800 μm [241,242,243,244]. Santos et al. [242] compared the size and structure of alginate aerogel beads formed using two different techniques, namely atomization and dripping followed by solvent exchange steps with and without solvent gradient before scCO_2_ drying. A mean particle diameter of 236 ± 3 µm with a high surface area (484 m^2^/g) was obtained for the atomization with exchange solvent gradient, while the aerogels prepared by dripping without gradient exchange of water to ethanol resulted in a larger mean particle diameter of 2796 ± 127 µm, having a surface area of 381 m^2^/g. The alginate aerogel beads that were prepared by the dripping method were further used for impregnation with trans-resveratrol under scCO_2_. The maximum loading capacity of resveratrol was 77%. The surface morphology of alginate aerogel particle and drug-loaded alginate particle is shown in Figure 15, where the resveratrol crystal formation around the aerogel bead is visually apparent. 

Lovskaya et al. [243] also applied the dripping method for the preparation of alginate aerogels. Its potential as drug delivery system was evaluated by supercritical adsorption impregnation of active substances (ketoprofen, nimesulide, loratadine), where the maximum loading capacity was observed for loratadine (31%). The aerogels and corresponding active substances were suggested to be in an amorphous state, and the study highlighted the stability of this state after six months of storage. In another study, microparticles of alginate aerogels (mean diameter: 100 μm) were impregnated with gallic acid and passion fruit bagasse extract, and studied after emulsion gelation, solvent exchange, wet impregnation and scCO_2_ drying [244]. The alginate solution concentration was varied (1, 2, and 3 wt%) resulting in surface areas between 516 and 698 m^2^/g, in turn affecting the maximum loading capacity of gallic acid. 

The preparation and use of porous alginates for water treatment applications have been reported in several recent studies [245,246,247,248]. Generally, freeze-drying has been used after ionic gelation and the characterization focused on the absorption capacity, rather than the structure and specific surface area that typically is lower compared to drying using scCO_2_ (53 to 320 m^2^/g). The adsorption capacity of methylene blue was 151 mg/g when alginate was combined with graphene oxide-montmorillonite [247], 323 mg/g for an alginate/gelatin/graphene oxide system [249], and as high as 446 mg/g when activated carbon was incorporated [248]. Wang et al. [250] prepared floatable magnetic alginates by combining with Fe_3_O_4_ and industrial alkaline residue, forming a cost-efficient and easily recyclable spherical material. The maximum adsorption capacity of Cd^2+^ was 39 mg/g. Alginate aerogels have previously shown a high selectivity toward Cd^2+^, compared to other metal ions, with a reported adsorption capacity as high as 177 mg/g [245]. 

Alginate is known as an inherently flame-retardant material, which makes it promising for the development into low flammability aerogels [251]. For insulation applications, alginate has been combined with other components to achieve lower thermal conductivity, or further suppress flammability. However, alginate is highly hydrophilic which restricts its use for insulation applications. 

The moisture resistance was shown to be improved by, for example, chemical vapor deposition of methyltrimethoxysilane after freeze-drying to produce ultralight (0.036 g/cm^3^), hydrophobic and flame-retardant porous alginate with low thermal conductivity (0.332–0.0165 W/mK, 25–1000 °C) and a surface area up to 146 m^2^/g [252]. Also, nanorod clay (palygorskite) was combined with alginate to achieve fire and heat resistance. Crosslinking has been used as another approach to improve water resistance of ice-templated alginate melamine-formaldehyde materials having thermal conductivities of 0.035–0.047 W/mK, depending on the ratio between alginate and melamine-formaldehyde [235].

Li et al. [253] applied a facile preparation procedure to assemble mechanically strong and flame-retardant alginate/clay porous materials by adding p-toluenesulfonic acid monohydrate to alginate/clay and water/sodium salt solution, to achieve pH 6 and pH 8, prior to ice-templating. By adjusting the pH (crosslinking), the morphological structure of the material changed from layered to network, and its robustness was improved (compressive moduli of 51 ± 7 MPa). The crosslinking further affected the combustion behavior by reducing the number of toxic products, but did not show an effect on the thermal stability of the material. 

In the view of sustainable processing, carbon dioxide induced gelation was studied as an alternative method to conventional gelation, where a suspension of calcium carbonate dispersed in a sodium alginate solution induces irreversible gelation without additional pH modifiers or crosslinkers [254]. By combining gelation, solvent exchange and supercritical drying into an integrated process, the processing steps could be reduced. The approach resulted in lightweight alginate aerogels (0.06 g/cm^3^) with a surface area of 545 m^2^/g and a mesopore volume of 6.98 cm^3^/g, combined with a thermal conductivity in the range of 18 to 22 mW/mK, making this method promising for thermal insulation applications. 

#### 3.2.2. Aerogels from Carrageenan

**Figure 16:**
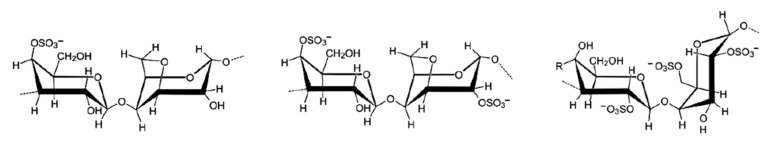
Repeating units in -κ, -ι and λ-carrageenans.

Carrageenans are polysaccharides with a pendant sulfated anionic group and are extracted from marine algae (Figure 16). Carrageenan is found within the cell membrane of seaweeds and within the intercellular matrix between the cellulosic fibers. Carrageenan consists of repeating units of galactose and 3.6-anhydrogalactose. These building blocks are linked by α1,4- and β1,4-glycosidic linkages. Depending on the sulphonation degree, three types of carrageenans can be distinguished including kappa (κ), lambda (λ), and iota (ι), which vary depending on the position and number of sulfate groups. The synthesis of gels and aerogels from carrageenans is based on “double-helix” formation for κ and ι carrageenans during the reversible thermally induced sol-gel transition. 

Carrageenan is widely used in diverse applications such as food, pharmaceutical and cosmetic industries. Due to their availability and surface properties, their broad functionality, low toxicity, biocompatibility, and biodegradability, carrageenan porous materials and aerogels are very promising in tissue engineering, drug delivery, environmental remediation catalysis, and many other applications [255,256,257,258,259,260,261,262,263,264,265].

**Figure 17:**
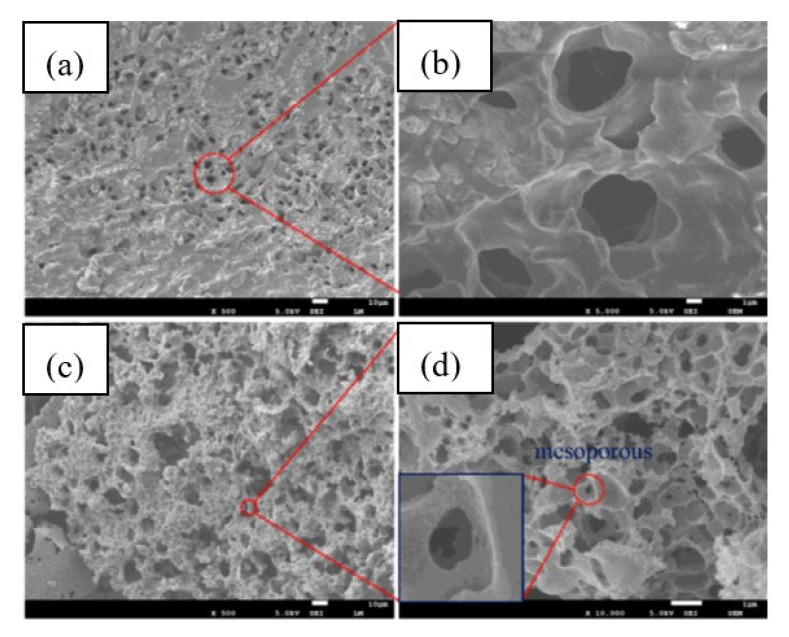
Different morphologies of freeze-dried lignosulfonate/κ-carrageenan (**a**,**b**) and their carbon counterparts (**c**,**d**) [264]. Reprinted from International Journal of Biological Macromolecules, 148, Lv, D.; Li, Y.; Wang, L., Carbon aerogels derived from sodium lignin sulfonate embedded in carrageenan skeleton for methylene-blue removal, 979–987, Copyright 2020, with permission from Elsevier.

Abdellatif et al. showed that freeze-drying of ι-carrageenan results in porous materials with a macroporous structure [260]. ι-carrageenan was crosslinked with polyamidoamine (PAMAM) using citric acid as a biodegradable crosslinker to produce porous carrageenans that contain multiple nitrogen atoms (Figure 17a). The incorporation of PAMAM and the use of freeze-drying generated ι-carrageenan materials with high specific surface areas from 130 to 400 m^2^/g, being efficient materials to remediate dyes and heavy metal ions from aqueous media.

A recent study investigated an ecofriendly way to make lignosulfonate/κ-carrageenan porous carbons [264]. The carrageenan formed a three-dimensional network with uniformly dispersed lignin sodium sulfonate (NaLS). Porous carbons were obtained after gel aging, freeze-drying, carbonization and activation (Figure 17b). An efficient dye adsorption of methylene blue (MB) was achieved due to the hierarchical porous structure having a specific surface area, microporous volume, and pore diameter around 600 m^2^/g, 0.3 cm^3^/g and 2.2 nm, respectively, with an adsorption capacity of 422 mg/g.

**Figure 18:**
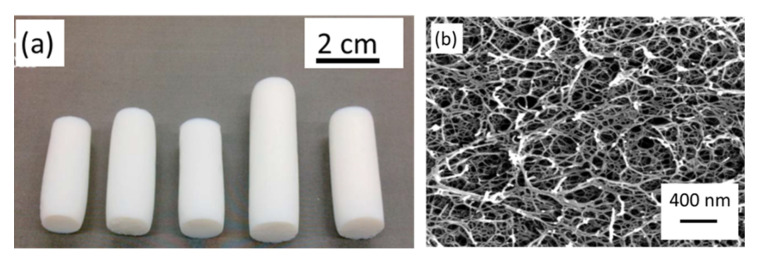
κ-carrageenan aerogels prepared via gelation with KSCN: (**a**) monoliths made from solutions of polymer concentration (from left to right) 1; 1.5; 2; 2.5 and 3 wt%, (**b**) scanning electron microscopy image of the fractured surface of aerogel made from 1 wt% solution (adapted from [257]).

K-carrageenan aerogels were made via gelation in KCl solution and oil absorption was studied [263,265]. The density of aerogels increased from 0.13 to 0.24 g/cm^3^ with the increase of carrageenan concentration from 0.4 to 2 wt%, respectively. Oil absorption by aerogels was investigated and stiff “oleogels” were suggested to be useful in food, pharmaceutical and cosmetic applications. Oil content was up to 80% and no oil release was recorded. K-carrageenan was also used to fabricate monolithic aerogels using potassium thiocyanate (KSCN) to induce gelation [257]. A variation in the concentration of κ-carrageenan and the concentration of specific ions influenced physicochemical properties such as shape, size, and density (Figure 18). The latter varied from 0.04 to 0.160 g/cm^3^ with the polymer concentration in solution being 0.5 to 3 wt%, respectively. The specific surface area was around 230 m^2^/g with no significant influence of κ-carrageenan concentration. 

A detailed study of the influence of various processing conditions such as the type and concentration of carrageenan and the type and concentration of crosslinker (K^+^, Ca^+^, Al^3^^+^) on the properties of carragenan aerogel beads was performed in ref. [263]. The type and concentration of crosslinker were shown to control aerogel properties. The density of aerogels varied from 0.06 to 0.5 g/cm^3^ and the specific surface area from 33 to 174 m^2^/g. 

### 3.3. Aerogels from Chitosan

**Figure 19:**
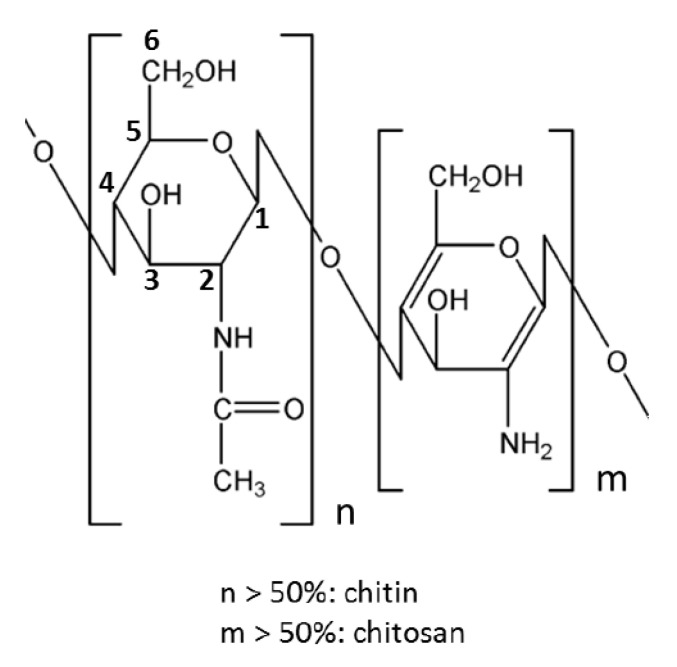
Structural formula of chitin and chitosan. Adapted from [266].

Chitosan is a pseudo-natural cationic polysaccharide obtained from chitin which is extracted from crustacean shells and some fungi. Chitin and chitosan are linear copolymers of d-glucosamine and *N*-acetyl-d-glucosamine linked by β (1 → 4) glycosidic bonds, as shown in Figure 19.

Chitin is the second most abundant natural biopolymer and exists in 3 polymorphic forms (α, β and γ-chitin) organized in microfibrils. Due to its high crystallinity, chitin is not soluble in common solvents and can only be solubilized in strong acids, organic solvents and in hot aqueous solution with a high concentration of salt (LiCNS, Ca(CNS)_2_, CaI_2_, CaBr_2_, CaCl_2_) [264].

To obtain chitosan from chitin, the acetyl groups must be removed. To this aim, two processes can be used: the Broussignac process [267] and the Kurita process [268]. In the Broussignac process chitin is added to a deacetylation reagent: a mixture of potassium hydroxide, ethanol and monoethyleneglycol. This solution is heated and when the desired degree of acetylation (DA) is reached, chitosan is filtered, washed, and dried. In the Kurita process, a suspension of chitin in an aqueous sodium hydroxide solution is heated to high temperature under a nitrogen stream. The time of reaction depends on the desired DA. 

Chitosan presents a DA below 50%. It is insoluble in water, alkaline solutions and organic solvents but can be solubilized in acidic media. Chitosan shows antibacterial, antiviral, and antifungal properties, which are ascribed to its amino group [269]. Two mechanisms are at the origin of antimicrobial properties: a decrease of the membrane permeability due to interactions between anionic groups at the surface of the cells with the cationic groups of the polymer chain; and the inhibition of the synthesis of the antimicrobial RNA because of bonding between chitosan and cellular DNA via the amino groups. This antimicrobial activity depends on molecular weight, DA, concentration, and the presence of the cationic amine group of chitosan.

Chitosan presents hemostatic properties by stimulating the formation of blood clots [270]. In a physiological environment, the amine group is protonated and interacts with red blood cells which are negatively charged, inducing clotting. Several wound dressings such as HemCon^®^ and Thermoguard^®^ are commercialized to stop hemorrhages. Chitosan-based materials are biocompatible, biodegradable, and non-allergenic. Chitosan also presents bioactivity by speeding up wound healing, stimulating the immune system and decreasing the cholesterol level.

The chitosan monomer presents several functions such as a primary amine as well as primary and secondary hydroxyl functions which can be chemically modified [271,272] without interfering with the degree of polymerization. These chemical modifications can improve the properties of chitosan for drug delivery, metal chelation and its hydrophilicity. Phosphorylation can be performed on all the mentioned functions, whereas thiolation can be done on the amine function. Crosslinking of chitosan is possible through the amine function with aldehydes like glutaraldehyde or through the primary hydroxyl with chlorine compounds like epichlorohydrin. Grafting onto chitosan has also been performed using a radical initiator (such as 2,2′-azobisisobutyronitrile or potassium persulfate), a redox system (like Fe^2+^/H_2_O_2_), and Ce(IV).

**Figure 20:**
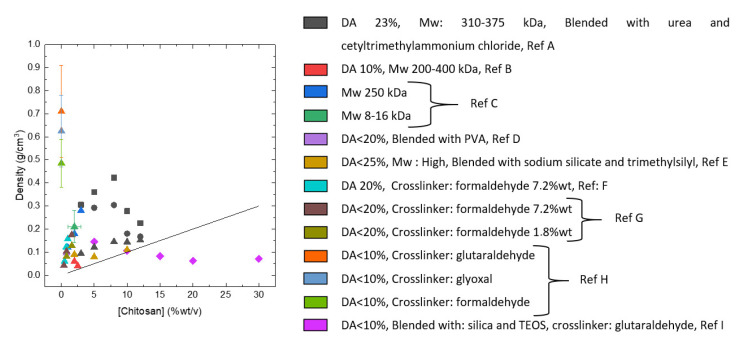
Variation of the density as a function of the chitosan concentration. Methods of drying are represented by squares for ambient drying, circles for drying at 65 °C, diamonds for freeze drying and triangles for supercritical drying. The solid line represents the density for pure chitosan materials without shrinkage and without mass loss. Ref A: [273], Ref B: [274,275], Ref C: [276], Ref D: [277], Ref E: [278], Ref F: [279], Ref G: [280], Ref H: [281], Ref I: [282].

Figure 20 shows the influence of chitosan concentration on the density of chitosan aerogels, some examples for chitosan dried in ambient conditions and via freeze-drying are also shown for comparison. The theoretical density calculated for the cases of an absence of shrinking is shown by a solid line. As expected, samples dried under ambient conditions have a relatively high density. Generally, the density of aerogels increases with the concentration. Some points are below the theoretical density, for which the authors did not propose any explanation.

**Figure 21:**
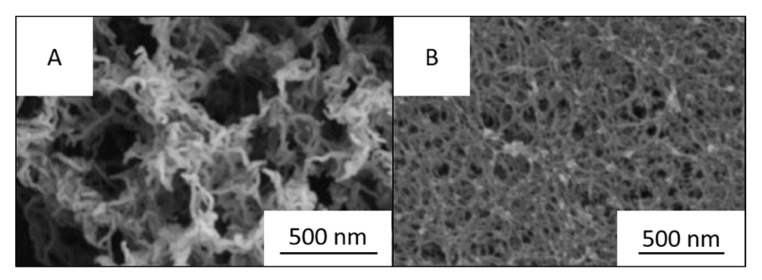
Morphology of chitosan aerogels (**A**) 2.5 w/v% chitosan, DA = 10%, MW 200–400 kDa, solvent 2%v acetic acid [275], and (**B**) 0.4 wt/v% chitosan, DA < 20%, solvent 2%v acetic acid, crosslinker formaldehyde 7.2 wt% [280]. (adapted with permission from Takeshita, S.; Yoda, S. Chitosan aerogels: transparent, flexible thermal insulators. Chemistry of Materials 2015, 27, 7569–7572. Copyright 2015 American Chemical Society.).

Two examples of chitosan aerogel morphology are shown in Figure 21. The networks are composed of entangled nanofibers of 5–10 nm in diameter with a broad porosity (mesopores and macropores). Both samples present a similar porosity and density, but the pores look smaller in image B. The specific surface area of the sample in image B [280] is higher than the one in image A [275], most probably due to the presence of a crosslinker.

**Figure 22:**
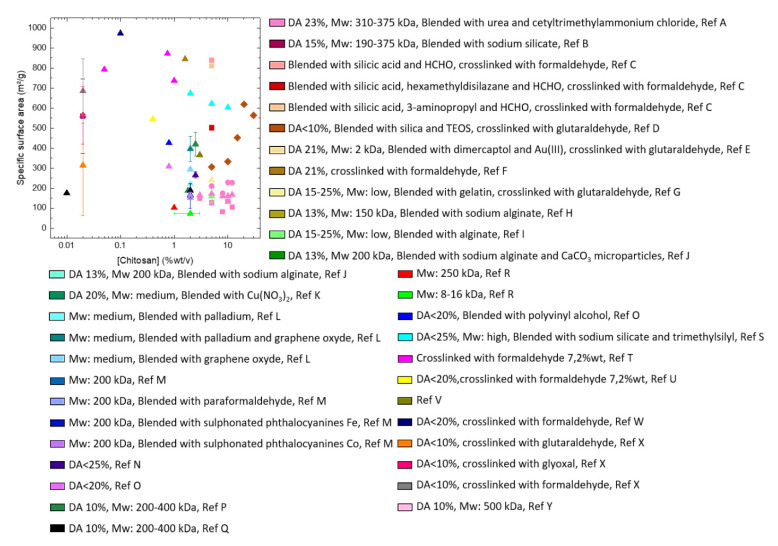
Influence of the chitosan concentration on the specific surface area for different drying methods. The shape of the symbol represents the drying method: square for ambient drying, circle for drying at 65 °C, triangle for drying under supercritical conditions and diamond for freeze drying. Ref A: [273], Ref B: [283], Ref C: [284], Ref D: [282], Ref E: [285], Ref F: [286], Ref G: [287], Ref H: [288], Ref I: [289], Ref J: [237], Ref K: [290], Ref L: [291], Ref M: [292], Ref N: [293], Ref O: [277], Ref P: [275], Ref Q: [274], Ref R: [276], Ref S: [278], Ref T: [279], Ref U: [280], Ref V: [294], Ref W: [295], Ref X: [281], Ref Y: [296].

The influence of the chitosan concentration on the specific surface area is shown Figure 22. It does not seem to depend on the chitosan concentration, but only on the presence of crosslinker and of another material. Crosslinker increases the number of pore walls inside the network, decreasing the pore size and increasing the specific surface area. Freeze dried samples obtained by Ma et al. show a very high specific surface area for this type of drying thanks to the presence of silica aerogel inside chitosan network [282]. The ice crystals did not break the pores during the drying, conserving the nanopores, and allowing for a high specific surface area.

**Figure 23:**
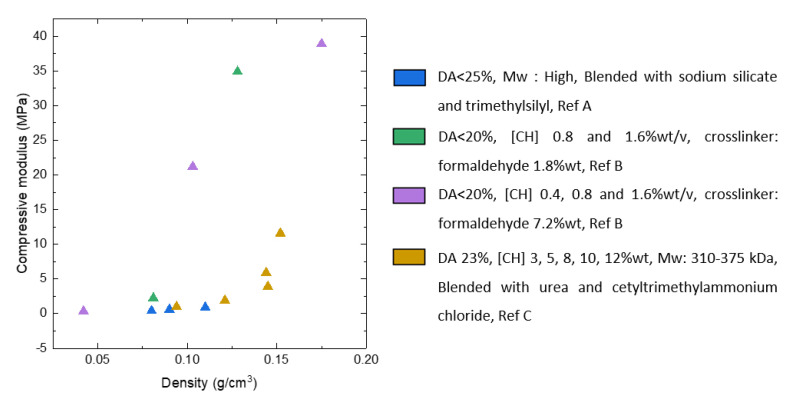
Evolution of the compressive modulus as a function of chitosan concentration. All materials are obtained by supercritical drying. Ref A: [287], Ref B: [278], Ref C: [280].

There are few data on compressive modulus for porous chitosan (Figure 23). Increasing the chitosan concentration seems to increase the compressive modulus. The presence of gelatine [287] reinforced the network leading to a higher compressive modulus than that of the neat chitosan aerogel: 165–181 kPa, 52 kPa, and 120 kPa for hybrid material, neat gelatine, and neat chitosan, respectively. This improvement can be explained by ionic interactions during gel formation leading to an interpenetrated network with gelatine and chitosan chains. The compressive modulus is shown (Figure 23) as a function of density. The higher the density, the higher the elastic modulus, as expected. The higher the amount of crosslinker, the higher the elastic modulus, as shown in ref [280]. 

**Figure 24:**
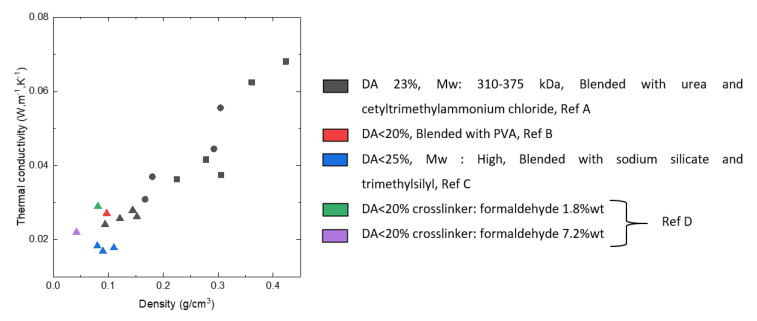
Evolution of the thermal conductivity as a function of density. Ref A: [273], Ref B: [277], Ref C: [278], Ref D: [280]. The shape of the symbol represents the drying method: square for ambient drying, circle for drying at 65 °C and triangle for drying under scCO_2_.

Thermal conductivity of chitosan aerogels as a function of density is shown in Figure 24. The lower is the density, the lower is the thermal conductivity. Till now, the classical U-shape curve known for silica aerogels for the conductivity vs density was not recorded for chitosan aerogels. Only materials dried under supercritical conditions showed thermal conductivity below that of air, which makes them super-insulating materials. 

Chitosan aerogels can potentially be used in various domains as an adsorbent [281], as a catalyst [297], as a thermal insulator [280] and in biomedical applications [286]. For the latter, chitosan is useful for its haemostatic and antibacterial properties in wound dressings. It can also be employed for tissue engineering and as drug delivery matrix. Chitosan aerogels present tunable properties and the possibility to perform chemical modifications. For example, the morphology can be tuned by blending with other organic or inorganic materials. This usually reinforces the network, leading to better mechanical properties, or adds new functionalities. Chemical modifications and blending allow achieving values of porosity and of specific surface area similar to or higher than those of neat chitosan aerogels.

### 3.4. Aerogels from Pectin

**Figure 25:**
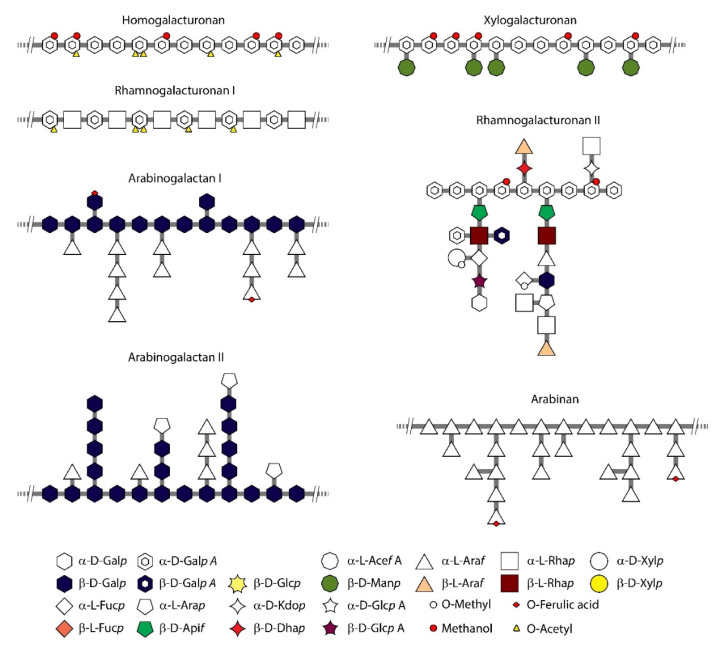
Schematic presentation of pectin structural elements (adapted from [298]).

Pectin is a heteropolysaccharide with a complicated and heterogeneous structure, depending on the source of its production (plant species, type of tissue or cell part) [298,299]. Pectins, also called pectic polysaccharides, contain a significant amount of galacturonic acid. Several different polysaccharides in the pectic group have been identified and characterized. A common feature in their structure is the presence of α-D-galacturonic acid molecules linked by glycosidic bonds between carbon atoms in the C-1 and C-4 positions. However, the differences concern the size of the molecules, the length and degree of branching of the chains, the composition of the sugars that make them, as well as the degree of methylation and acetylation. It is estimated that pectin contains no less than 17 different monosaccharides linked by at least 20 different bonds [298,300]. The most important polysaccharides forming the pectin structure are: homogalacturonan (HG), rhamnogalacturonan I (RG-I), rhamnogalacturonan II (RG-II), xylogalacturonan (XGA), arabinan, arabinogalactan I, and arabinogalactan II (Figure 25) [298,301]. 

**Figure 26:**
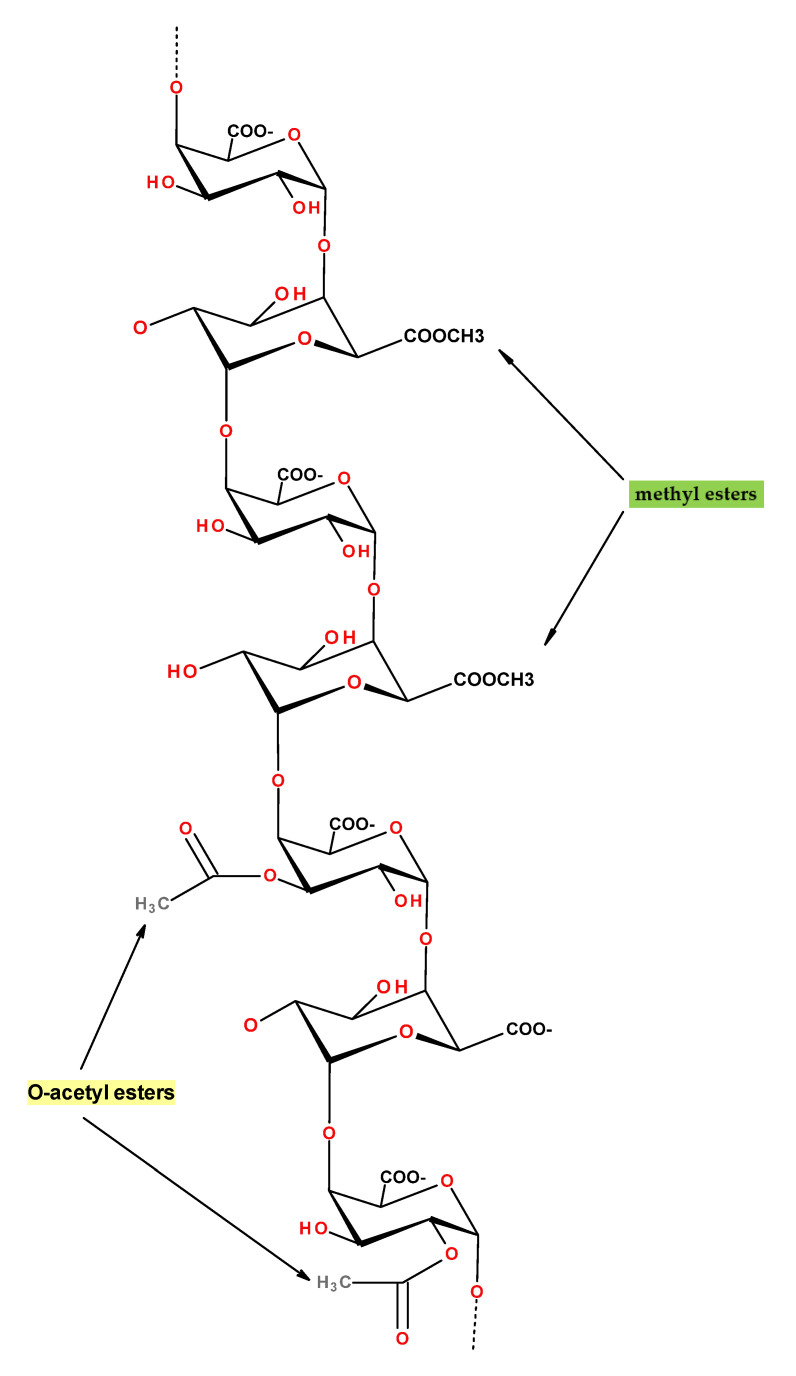
Schematic presentation of the primary structure of homogalacturonan.

In the pectin chain HG and RG are arranged alternately, thus forming regions consisting of branched “hairy” chains (made of type I rhamnogalacturonan units) and ‘‘smooth” linear, homogeneous regions (made of homogalacturonan units) [301,302,303,304]. According to the definition given by the European Commission and the Food and Agricultural Organization (FAO)/World Health Organization (WHO) Expert Committee on Food Additives, pectin should contain at least 65% of galacturonic acid, i.e., homogalacturate [305]. HG is the dominant type of polysaccharide that forms the structure of the pectin molecule. It accounts for about 60–65% of all pectins building the structures of plant tissues. The HG chain consists of approx. 70–100 residues of 1,4-linked α-D-galactopyranosyluronic acid (Gal*p*A) which can be partly methylesterified at carbon C6 or O-acetylesterified at oxygen O2 or O3 (Figure 26) [301,306,307]. The average molar weight of HG is not very high and, depending on the raw material source, it may amount to 16,000–43,000 Da [308].

Pectin is a structural heteropolysaccharide present in the cell walls (middle lamella, the primary and secondary) of many plants [300,309]. Usually, commercial pectins are extracted mainly from citrus peels (25–30% of the dry matter) or apple pomace (15–18% of the dry matter) and are widely used in the food industry as gelling and thickening agents. Currently, pectins from other sources such as mango, sugar beets or sunflower heads are also being increasingly used [310,311,312]. In the biomass, this polysaccharide is commonly connected with other cell wall components such as cellulose, hemicellulose and lignin [313].

Pectins are usually extracted by chemical (alkaline, acid, or mixed) and enzymatic methods [314]. The alkaline method preserves the neutral sugar side chains in pectin, but the pectin’s methyl ester and acetyl groups are hydrolyzed in a β-elimination reaction [315]. The enzymatic extraction method takes more time than chemical methods, but it allows to obtain pectins with a higher molecular weight and degree of esterification (DE) [316]. Moreover, it can reduce the emission of waste acids or alkaline solutions to the environment [317]. A typical pectin production process involves hot acid extraction (pH ~2.0) from raw plant material (citrus peel, apple pomace, potato, sugar beets, cocoa husk etc.) [318,319]. Most pectins are extracted using sulfuric acid and hydrochloric acid while maintaining the pH in the range of 1.2 to 5. Mixed chemical extraction could be carried out with acid (sulfuric acids) and with reagents such as sodium chloride, EDTA, glycerol, sodium polyphosphate or ammonium sulphate [320]. Finally, the microwave extraction of pectin from fruit peel is also used [321,322]. This unusual extraction method, along with the ultrasonic method, are together classified as ecological extraction methods [323,324]. The resulting polymer is a commercial, highly methylated (HM) pectin, with a degree of esterification of about 70%. Additional demethylation processes are used to obtain other types of pectin.

Pectin shows gelling properties according to “egg-box model” (Figure 13b), as well as thickening and emulsifying properties, that have been used for a long time in the food industry (production of fruit preserves, confectionery and dairy products, ketchup, and mayonnaise) [325,326]. Currently, pectins are also used as ingredients of jellies, acidophilic milk drinks, margarines, salad dressings, ice creams, or coatings of products intended for frying and as a factor limiting the amount of absorbed fat [327]. Pectins are also used in pharmaceutical and medical products [328,329] and the cosmetics industry [320], where their biochemical reactivity, availability, easy isolation and non-toxicity are exploited. The presence of pectins in diet has a positive effect on the gastrointestinal microflora [330,331] as well as the glucose [332,333], cholesterol and lipid [333,334] metabolism. Moreover, the detoxification and anti-carcinogenic properties of these polysaccharides have been proven [330,335]. In recent years, it has been reported that pectin derived from sickle cell disease (*Bupleurum falcatum*) may be used in the prevention and treatment of gastric ulcers [336]. The anti-inflammatory effect of pectins has also been confirmed [337,338].

Pectin aerogels are generally obtained by dissolving the polymer in water, solutions can be gelled or not, followed by solvent exchange and ScCO_2_ drying [339]. Pectin DE, solution pH and cation concentration (if any) significantly affect the final properties of pectin aerogels. For example, pectin aerogels produced by thermal and acid gelation have specific surface area of 485 m^2^/g (powder) and of 200 m^2^/g (monoliths) [340,341]. Pectin concentration in solution usually varies from 2 to 6 wt% and density is from 0.03 to 0.2 g/cm^3^. One can find examples of pectin aerogels produced from low methoxyl (LM) pectins [341,342] as well as high methoxyl (HM) pectins [33,343,344,345]. It has also been found that galacturonic acid amidation improves the LM pectin gelation process reducing the amount of calcium ions consumed [342]. 

The choice of the preparation process significantly affects the properties of pectin aerogels. There are many reports in the literature on the various ways of obtaining pectin aerogels, e.g., with the use of direct gelation in an acidic environment [33] or ionic gelation with the use of different types of cations (calcium, zinc, strontium) at various concentrations [342,344,346,347]. Another type of process is non-solvent induced phase separation [341,348,349,350,351]. Currently, there are more and more works describing the preparation of pectin-based composite aerogels, for example: pectin–xanthan aerogel [350], alginate–pectin system [234,352,353], polyaniline–pectin aerogel [351], melamine–formaldehyde-pectin aerogel [354], pectin–silica aerogel [346,355], and with micro-and nanoparticles such as TiO_2_ [15], clay [356], magnetic nanoparticles [343], or boron nitride nanosheets [357].

**Figure 27:**
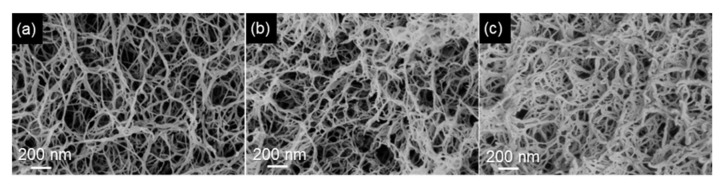
SEM images of LM pectin aerogels from 2 (**a**), 4 (**b**) and 6 wt% (**c**) solutions at pH 2 cross-linked with calcium [16]. Reprinted from Carbohydrate Polymers, 196, Groult, S.; Budtova, T., Thermal conductivity/structure correlations in thermal super-insulating pectin aerogels, 73–81, Copyright 2018, with permission from Elsevier.

**Figure 28:**
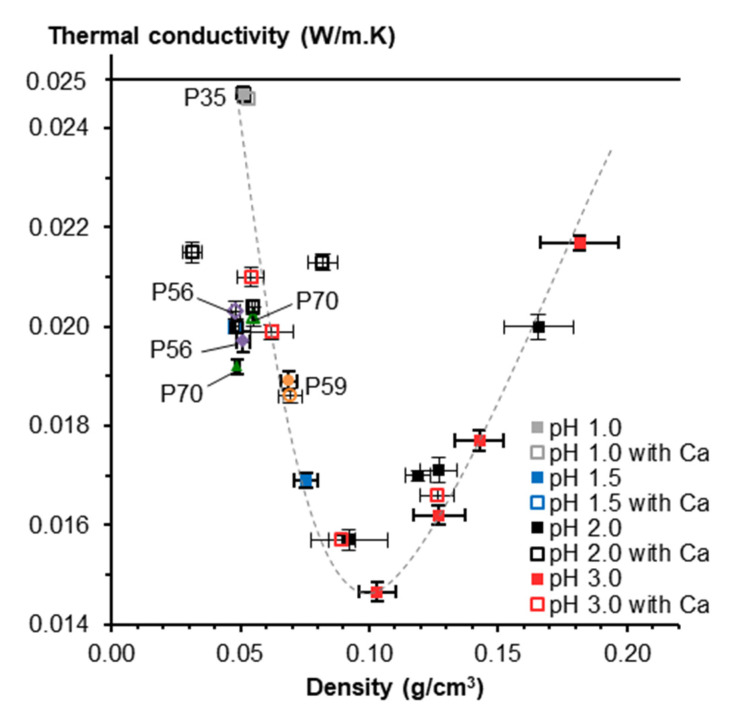
Thermal conductivity of LM pectin [16]. Reprinted from Carbohydrate Polymers, 196, Groult, S.; Budtova, T., Thermal conductivity/structure correlations in thermal super-insulating pectin aerogels, 73–81, Copyright 2018, with permission from Elsevier.

Pectin aerogels have a homogenous fibrillar network morphology with a pore size ranging from 50 to 300 nm in diameter (Figure 27) [339], and a large specific surface area (typically from 270 to 600 m^2^/g) [16,342]. Moreover, it was shown that aerogels with a high proportion of macropores, low density of about 0.05 g/cm^3^ and a rather low specific surface area, about 300 m^2^/g, are obtained from strong ionic gels formed in the presence of calcium ions (around pK_a_ of pectin) which “resist” shrinkage during solvent replacement and drying [339]. Pectin aerogels with similar morphology and density were obtained at low pH. Some pectin aerogels have also been found to be thermal super-insulating materials due to their small pore size and low density (Figure 28) [16]. Their thermal conductivity has been shown to be in the range of 0.015–0.022 W/mK [16,33,349,358]. Since thermal conductivity is a very sensitive parameter reflecting the morphology of the aerogel, it can significantly change in hybrid aerogels. For example, it was shown that polyaniline–pectin aerogels had a thermal conductivity in the range of 0.033–0.038 W/mK [351].

It was also shown that doping of pectin aerogels significantly influences their mechanical properties and morphology as the introduction of clay (sodium montmorillonite) into pectin aerogel increased its compression modulus [357]. In the case of aerogels prepared from 5 wt% pectin solutions, the modulus increased from 70 kPa to 330 kPa after adding 2.5 wt% clay. Furthermore, Yang et al. [357] investigated the enhancing effect of boron nitride nanosheets (BNNSs) on pectin-based aerogels; the modulus increased from 91 kPa to 199 kPa after adding BNNs at mass ratio BNNSs/pectin 1:10. 

Due to their unique properties and high biodegradability, pectin aerogels and their composites can be widely used in various industrial sectors. Thanks to their small pore size and low density, some aerogels are thermal super-insulating materials (Figure 28) [33,358,359]. However, protection against aging is needed. Moreover, it was found that porous aerogels composed of pectin and clay have general mechanical properties similar to rigid polyurethane foams, which makes them promising materials for various engineering applications [356]. Due to their non-toxicity and biocompatibility, pectin aerogels could be used as packaging and edible coatings for food protection [15,20]. Pectin aerogels, due to their polyelectrolyte nature and sensitivity to pH, have gained special attention in biomedical applications. This type of aerogel could be used as carrier for controlled drug release [343,344,360]. Due to the ability of pectin aerogels to release substances upon a change in pH, it is believed that they can be used as carriers for the delivery of drugs by the oral or mucosal route in the body (change from the acid gastric medium to the neutral intestinal environment) [342,345]. Considering that pectin can be extracted from food waste, the production of pectin aerogels fits perfectly into the biorefinery concept [323]. 

### 3.5. Aerogels from Starch

**Figure 29:**
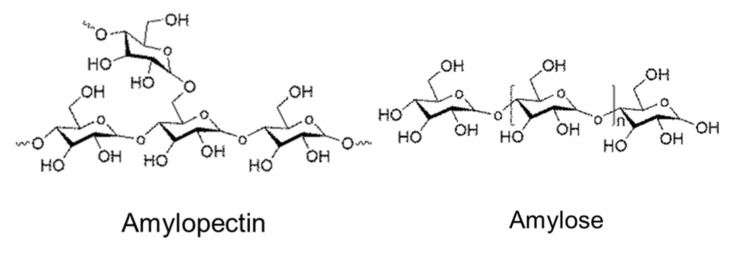
Chemical structures of amylose and amylopectin.

Starch is one of the most abundant natural polysaccharides which can be found, for example, in corn, potato, wheat and rice. Starch is composed of two main polymers: linear amylose and branched amylopectin (Figure 29). Amylose is linear (1 → 4)-α linked glucose and amylopectin is (1 → 4)-α-linked D-glucose with (1 → 6)-α branches. Amylose and amylopectin are organized in semi-crystalline granules with a diameter from few to few hundreds of microns, depending on the starch source. The ratio of amylose to amylopectin also depends on the plant type and can vary from zero amylose (waxy starches) up to 80% amylose (high amylose corn). In addition to food and feed, starch is used as an additive in paper and textile [361], to fabricate Pickering emulsion system [362], and to make biodegradable films and foams for packaging [363].

Starch aerogels are prepared via dissolution-retrogradation (to form gel)-solvent exchange-supercritical CO_2_ drying. The first starch aerogels based on wheat, corn and high amylose corn starches were named “microcellular foam” by Glenn et al. in 1995 [364]. By adjusting the processing methods, starch aerogels can be made in the shape of monoliths [36] or as particles, the latter usually via emulsion-gelation techniques [44,365]. Composite starch aerogels can be prepared by mixing starch solution with other components [366]. 

There are many parameters which affect the final properties of the starch aerogels. The main ones are starch concentration [43,367,368], starch source [36,368], gelatinization temperature [43], and supercritical drying conditions [365] (such as pressure, temperature, CO_2_ flow rate, and depressurization rate etc.). García-González et al. reported that below 7 wt% of corn starch it was not possible to form “self-standing” gels, and above 15 wt% the viscosity was too high [367]. The increase in starch concentration from 7 to 15 wt% decreased the volume shrinkage from 49% to 25% [367]. 

**Figure 30:**
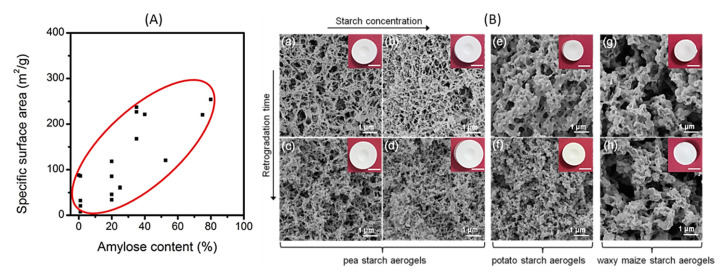
(**A**) Specific surface area of monolithic starch aerogels as a function of amylose content [36,43,44,368,369] and (**B**) SEM images of starch aerogels from various sources at different initial starch concentrations and retrogradation times: (**a**) pea-5wt%-1day-aerogel, (**b**) pea-8wt%-1day-aerogel, (**c**) pea-5wt%-4day-aerogel, (**d**) pea-8wt%-4day-aerogel, (**e**) potato-11wt%-1day-aerogel, (**f**) potato-11wt%-4day-aerogel, (**g**) waxy maize-11wt%-30day-aerogel, (**h**) waxy maize-11wt%-45day-aerogel. The scale bar on SEM images is 1 μm, and on the photos of aerogels (insets) is 1 cm [368]. (Reprinted from Carbohydrate Polymers, in press, Zou, F.; Budtova, T., Tailoring the morphology and properties of starch aerogels and cryogels via starch source and process parameter, Copyright 2020, with permission from Elsevier).

Amylose plays an important role in the structure formation of starch gels as it crystallises faster than branched amylopectin. The shrinkage, density, and specific surface area of starch aerogels depend on the amylose content [36,368]. For example, the density decreases from 0.2 g/cm^3^ to 0.14 g/cm^3^ when the amylose content increases from 0% to 80% [36]. The specific surface area also depends on the amylose content: the higher the amylose content, the higher the specific surface area (Figure 30). As reported by Druel et al., the increase of amylose content leads to a triple increase in specific surface area, from 88 m^2^/g for waxy potato starch aerogels (amylose content 0%) to 254 m^2^/g for high amylose corn starch aerogels (amylose content 80%) [36].

Starch gelatinization and retrogradation also influence aerogel properties. A higher gelatinization temperature leads to a lower density and a higher specific surface area of the final starch aerogels [43]. The reason is that the high gelatinization temperature reduces the number of remnants of starch granules which do not “participate” in mesoporosity. In the same way, a higher mixing rate, at a fixed temperature, also leads to a higher specific surface area. Increasing the retrogradation time results in a lower specific surface area due to the increase of crystallinity and thicker pore walls [36].

The influence of supercritical CO_2_ drying conditions on starch aerogel properties was also studied [365]. Numerous parameters, such as CO_2_ flow rate, temperature, pressure, drying time and depressurization rate, can be varied. A higher CO_2_ flow rate leads to a lower specific surface area due to the expanded liquid in pores with a high dissolution of scCO_2_ in the solvent [365]. An increase in drying temperature results in a minor increase of the specific surface area [43]. Despite the fact that the increase of pressure increases the miscibility of CO_2_ with ethanol, the density of this mixture is increased leading to the decrease of diffusion and decrease of the specific surface area [43]. Drying time depends on the shape and dimensions of the sample; obviously, smaller samples need shorter drying times than monolithic blocks [367]. 

**Figure 31:**
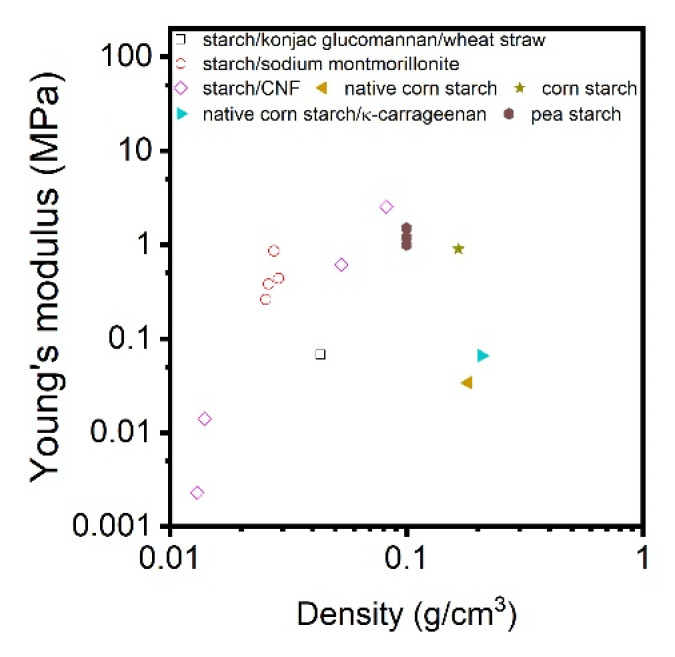
Compressive modulus of various porous starches and starch composites: dried with supercritical CO_2_ (filled points) and freeze dried (open points) [36,366,370,371,372,373].

Only a few studies investigate the mechanical properties of starch aerogels. Glenn et al. reported a compressive modulus of 21 and 8.1 MPa for aerogels from wheat starch (28% amylose) and from high amylose corn starch (70% amylose), respectively [364]. Recently, Druel et al. reported that the compressive modulus of starch aerogel increases with retrogradation time due to the increased crystallinity and the formation of thicker pore walls [36]. A comparison of the compressive modulus of freeze-dried and supercritically dried starches is presented in Figure 31. The Young’s modulus of starch-alginate aerogels was affected by the depressurization rate: an increase in the depressurization rate from 0.1 to 30 bar/min increased the modulus from 0.52 to 1.35 MPa [370].

Mixing starch with other components may significantly influence the mechanical properties of starch-based aerogels. The presence of melamine-formaldehyde improved the compression strength of starch-based aerogels as compared with melamine-formaldehyde aerogels [374]. Incorporation of zein to corn starch improved the Young’s modulus compared to neat corn starch-based aerogels [371]. 

**Figure 32:**
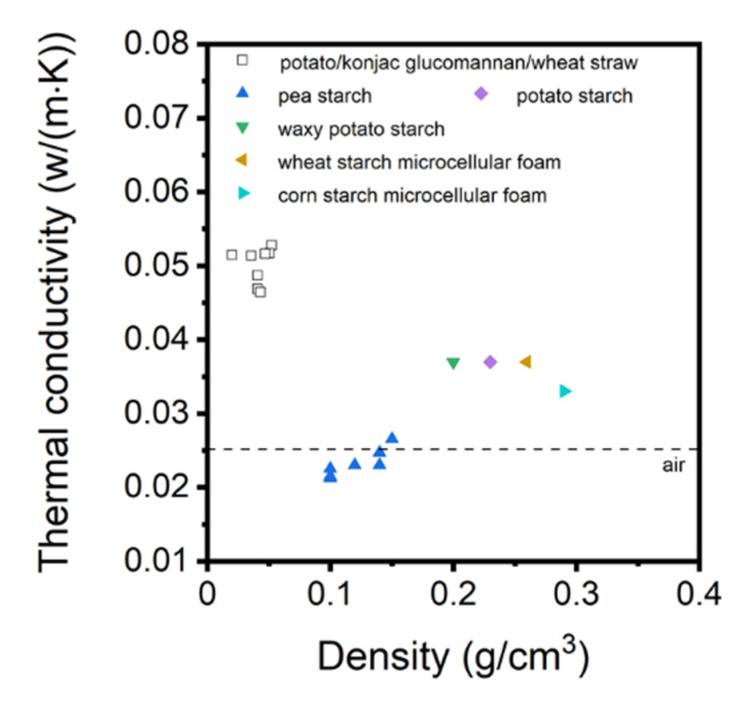
Thermal conductivity of various monolithic porous starch-based materials obtained via supercritical CO_2_ drying (filled points) and freeze drying (open points) [36,364,372].

The thermal conductivity of starch aerogels was first reported by Glenn in 1995, it varied from 0.024 to 0.044 W/m·K [364]. Druel et al. reported the influence of starch source and retrogradation time on the thermal conductivity of starch aerogels in detail [36]. Pea starch aerogels had the lowest thermal conductivity, around 0.021–0.023 W/m·K. These values were lower than those of aerogels based on waxy potato starch, regular potato starch and high amylose corn starch. The reason is that pea starch has the optimal amylose to amylopectin ratio leading to aerogels with the lowest density and finest microstructures. A long retrogradation time resulted in a higher thermal conductivity due to the increased crystallinity and thicker pore walls. The way of drying (freeze-drying or with supercritical CO_2_), processing parameters, and starch source influence porous starch thermal conductivity (Figure 32).

Until now, the major application suggested for starch aerogels is to use them as a matrix to encapsulate and release a drug. Encapsulation can improve the solubility [375,376] and bioaccessibility of a drug [369,377]. For example, encapsulation in a starch aerogel through supercritical CO_2_ impregnation prevented the formation of large crystals of phytosterol. The formation of thin platelate nanoparticles of phytosterol resulted in a 37-fold increased solubility in water as compared to crude phytosterols [378]. The impregnation capacity and morphology of phytosterol were also affected by the starch type and the shape of starch aerogels [369]. Corn starch aerogels had a higher impregnation capacity than wheat starch aerogels due to a higher specific surface area and pore volume. In vitro and in vivo release studies showed that encapsulation of the drug into starch aerogels enabled controlled release. For example, the impregnation of ketoprofen in starch aerogels or starch/poly(ε-caprolactone) aerogels scaffolds slowed down drug release in comparison with pure ketoprofen [379].

Starch based aerogels can be used as a template to make novel materials. Starch based aerogels and titanium isopropoxide were placed in an autoclave, where diffusion of titanium isopropoxide in supercritical CO_2_ into the surface of starch aerogels resulted in TiO_2_/starch hybrid aerogels. After heating at 500 °C in air for 5 h, starch was burned, and the remaining TiO_2_ network was a fine replica of the structures of the starch-based aerogels [380]. Starch based aerogels can also be used as a template to form conductive porous materials, such as poly(3,4-ethylenedioxythiophene) (PEDOT) networks for biomedical applications [366,381].

The low thermal conductivity of starch aerogels, as described above, shows that they can be used as thermal superinsulation materials [36,372]. Starch aerogels or their composites can also be used as fire-resistant materials [373]. Furthermore, Anas et al. reported that starch aerogels can be used as CO_2_ adsorption materials [382]. The heat of adsorption was determined by varying the pressure with temperature at a constant excess uptake. The results showed that wheat starch aerogel had the highest value compared to silica, resorcinol-formaldehyde, and carbon aerogels. By adsorbing antioxidant and antimicrobial ingredients such as quercetin, the application of starch aerogels can also be extended to active packaging applications [363].

### 3.6. Aerogels from Proteins

Proteins are another huge source of natural polymers that can potentially be used for making aerogels. Several vegetal and animal protein sources are available, such as whey, soy, potatoes, seeds, egg white, gliadin, collagen, keratin, and fish, offering an impressive list of natural matter for making porous materials, including aerogels. Proteins provide a wide range of potential functional groups such as –COO^−^, –NH_2_ and –SO_4_ [383]. However, literature on supercritically dried protein gels with high specific surface area is rather scarce [384,385,386,387,388,389,390,391,392]: the majority of works is devoted to freeze-dried protein gels with low specific surface area and the majority of applications are in the biomedical area (see, for example, ref [393]).

**Figure 33:**
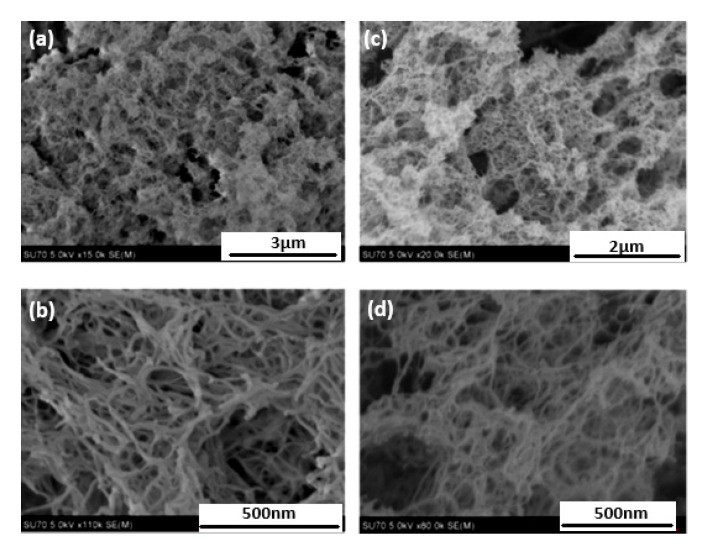
SEM images of an unloaded silk fibroin aerogel (**a**,**c**) and aerogel loaded with ~21 wt% ibuprofen (**b**,**d**). Each set of images are for the same sample at two different magnifications [390]. Reprinted from The Journal of Supercritical Fluids, Michael A. Marin, Rajendar R. Mallepally, Mark A. McHugh, Silk fibroin aerogels for drug delivery applications, 84–89, Copyright 2014, with permission from Elsevier.

Silk fibroin was used to make aerogels; it is usually dissolved in 10–15 M aqueous LiBr at 60–65 °C for several hours followed by dialysis. Gelation occurs through a heating process in acid media above the denaturation temperature of the protein, here, around 40 °C. The silk fibroin aerogel was loaded with ibuprofen under scCO_2_ and drug release kinetics were studied [390]. Aerogel density was 0.06 g/cm^3^ and specific surface area around 420 m^2^/g (Figure 33). Silk fibroin was also used to make hybrid aerogels with polymethylsilsesquioxane, a silane coupling agent was used to bind the two components and improve aerogel mechanical properties [392]. Neat silk fibroin aerogels’ specific surface area was around 400 m^2^/g, and it was more than doubled in the interpenetrated aerogel network with polymethylsilsesquioxane. Hybrid aerogels were tested for selective absorption of organic pollutants from water; the absorption capacity was 500–2600% (g/g). Silk fibroin hybrid aerogels showed excellent bending flexibility and acted as a high-performance filter for continuous water/oil separation [392].

The preparation of whey protein aerogels and freeze-dried gels was reported [384]; they were tested for absorbing oil for making oleogels. Whey and egg white protein were used to make monolithic and particular aerogels [388,391]. By controlling the pH and ionic strength of the solution during the gelation process, the porosity and surface area of the egg protein aerogel could be varied. The formation of smaller pores due to better crosslinking of protein molecules is promoted by a higher degree of denaturation of the egg white protein, increasing the volume of mesopores and small macropores [388]. The specific surface area of egg white protein aerogels was slightly lower (220–370 m^2^/g) than that of whey protein aerogels (380–420 m^2^/g), the latter did not depend on solution pH [388]. An increase in the sodium chloride concentration in the solution of egg white protein decreased the specific surface area of aerogels, especially at pH 2 and 11.5 [388].

Alatalo et al. used soy protein (as a nitrogen source and structure directing agent) mixed with cellulose or glucose (as carbon source) to produce carbon aerogels via hydrothermal carbonization [389]. The fabricated carbon aerogels were tested as cathode oxygen reduction reaction catalysts and as supporting material for a platinum catalyst [389]. The specific surface area of the starting freeze-dried glucose-soy protein and cellulose-soy protein materials was around 20 m^2^/g, and it significantly increased for their carbon counterparts, up to 450 and almost 700 m^2^/g, respectively. 

Potato protein isolate can also be used for the production of aerogels. Patatin aerogels present surface areas of 450, 180, and 350 m^2^/g at pH 3, 6, and 8, respectively [386]. 

Using natural proteins for aerogel production contributes to an increase in the sustainability of the overall food supply chain and the requirements of a circular economy [394], owing to their availability as a by-product of the food processing industry or waste streams. 

### 3.7. Aerogels from Organic Acids

Low molecular weight organic acids are a potential source of aerogels, but till now it remains practically unexploited, they are mainly used as crosslinkers or catalysts. One of the potential problems in making self-standing aerogels is weak mechanical properties of the network. An example of using a low molecular weight compound for making aerogels was reported in ref. [395]. 2,3-didecyloxyanthracene was used to make gels via two routes: gelation in ethanol and gelation directly in CO_2_ [395]. Very low density (0.002–0.006 g/cm^3^) friable aerogels were obtained. The specific surface area was low, at around 10 m^2^/g. 

Amino acid-based (phenylalanine and leucine, dissolved in toluene or tetralin) hydrophobic supramolecular aerogels were reported in ref. [396]: the density was 0.004–0.03 g/cm^3^ and the specific surface area around 90 m^2^/g. Interestingly, a low thermal conductivity, 0.026–0.027 W/m·K, was obtained. 

## 4. Conclusions

Nature provides us with an incredible amount of various matter and materials—they all have specific properties targeted to certain “applications”, and nothing is useless. It is well admitted that we still need to learn a lot from nature to use its ingredients in an efficient way. Bio-aerogels are very “young” materials and, until now, research has not focused on using the biorefinery approach. However, bio-aerogels are not an exception in terms of our inefficient use of biomass, in particular, for making materials. Only recently has the biorefinery approach started to penetrate industry, and this process is slow as it requires not only investments, but also another way of thinking.

This review presented an overview of various types of biomass used for making aerogels, with several examples of their properties and applications, including biomedical and environmental components. The reader would certainly have noticed that some sections are more developed than others, and this is reflecting the current situation. There is still a long way to go in applying the biorefinery approach to aerogels. The authors hope that this article will stimulate further research in this direction.

## Figures and Tables

**Table 1:** Examples of functionalized porous cellulose absorbents for oil-water treatment.

Cellulose Source	Modification Type	Absorbed Substance	Absorption Capacity, g/g	Cycles	Contact Angle	Reference
Coir fibers	Trimethylchlorosilane, Hexamethyldisilazane	Lubricant oil	10	N/A	148°, 140°	[195]
Mango wood scraps	CNF; CNF/PVA composite aerogel, esterification with stearic acid chloride	Various oils, organic solvents	58–65 35–95	15	159°	[190]
Bark of Abutilon theophrasti	CNF, Chitin, Cationic guar gums; Methyltrichlorosilane	Corn oil, organic solvents	6.8 9.4–21.9	>10	155°	[199]
Sisal leaves	In-situ Cu nanoparticles	Various oils, organic solvents	92–100 67.8–164.5	>10	150.3°	[191]
Cotton cellulose	Esterification with octanoyl chloride; crosslinking with hexamethylene-diisocyanate	Various oils, organic solvents	49.9–55.8, 40.8–48.7	N/A	138.7°	[192]
Hardwood cellulose pulp	1,4-butanediol diglycidyl ether, epoxidized soybean oil	Crude oil, Engine oil, Pump oil	37, 30, 28	30	132.6°	[193]
Canola straw	Hexadecyltrimethoxysilane	Motor oil, Sunflower oil	79, 162	20	139°	[198]
Paper waste; cotton fibers	Methyltrimethoxysilane	Various oils, organic solvents	68–78, 40–94	5	142.8°	[197]
Pinus elliottii	Methyltrimethoxysilane	Petroleum	68.4	N/A	119.85°	[196]

**Table 2:** Comparison of functionalized cellulose aerogel adsorbents for CO_2_ adsorption.

Cellulose Source	Modification Type	CO_2_ Adsorption Amount, mmol/g	Cycles	Reference
Microcrystalline cellulose (MCC, officinal class)	*N*-(2-Aminoethyl)(3-aminopropyl)methyldimethoxysilane	1.59	5	[202]
Cellulose powder (C, α phase, ≤25 μm),	3-aminopropyltriethoxysilane	1.20	20	[203]
Birch Kraft pulp	Phthalimide (1,3-dihydro-1,3-dioxisoindole)	5.20	N/A	[204]
CNF hydrogel	*N*-(2-Aminoethyl)(3-aminopropyl)methyldimethoxysilane	1.02 chemical 0.35 physical	N/A	[206]
Eucalyptus pulp	*N*-(2-Aminoethyl)(3-aminopropyl)methyldimethoxysilane	1.78	10	[205]

**Table 3:** Comparison of functionalized cellulose aerogel adsorbents for dyestuff removal.

Cellulose Source	Modification Type	Adsorbed Substance	Adsorption Capacity, mg/g	Cycles	Contact Angle	Reference
Kraft pulp	Methyltriethoxysilane (MTES)	Crystal violet dye	150	N/A	~143°	[210]
Microcrystalline cellulose (MCC, with a trade name of C10583)	Dopamine (DA)	Methylene blue dye	110	N/A	N/A	[208]
Cotton linter pulp	Zeolitic imidazolate framework (ZIF-67);	Methyl orange dye	617	N/A	N/A	[211]
Wood pulp CNC	Poly (methyl vinyl ether-alt-maleic acid) (PMVEMA), Polyethylene glycol (PEG)	Methylene blue dye	116.2	5	N/A	[220]
Microcrystalline cellulose (MCC)	Phenolic resin (PF); carbonization	Methylene blue dye	610.85	>4	N/A	[221]
Cellulose nanofibrils (CNF)	Graphene oxide (GO), Tetraethylorthosilicate	Methylene blue dye	608.4	N/A	N/A	[222]
